# Neuroinflammation is induced by tongue-instilled ZnO nanoparticles via the Ca^2+^-dependent NF-κB and MAPK pathways

**DOI:** 10.1186/s12989-018-0274-0

**Published:** 2018-10-19

**Authors:** Huimin Liang, Aijie Chen, Xuan Lai, Jia Liu, Junrong Wu, Yiyuan Kang, Xinying Wang, Longquan Shao

**Affiliations:** 1 Nanfang Hospital, Southern Medical University, Guangzhou, 510515 China; 2 Guangdong Provincial Key Laboratory of Construction and Detection in Tissue Engineering, Guangzhou, 510515 China; 3 Zhujiang Hospital of Southern Medical University, Guangzhou, 510515 China

**Keywords:** Zinc oxide nanoparticles, Taste nerve, Neuroinflammation, Calcium ion

## Abstract

**Background:**

The extensive biological applications of zinc oxide nanoparticles (ZnO NPs) in stomatology have created serious concerns about their biotoxicity. In our previous study, ZnO NPs were confirmed to transfer to the central nervous system (CNS) via the taste nerve pathway and cause neurodegeneration after 30 days of tongue instillation. However, the potential adverse effects on the brain caused by tongue-instilled ZnO NPs are not fully known.

**Methods:**

In this study, the biodistribution of Zn, cerebral histopathology and inflammatory responses were analysed after 30 days of ZnO NPs tongue instillation. Moreover, the molecular mechanisms underlying neuroinflammation in vivo were further elucidated by treating BV2 and PC12 cells with ZnO NPs in vitro.

**Results:**

This analysis indicated that ZnO NPs can transfer into the CNS, activate glial cells and cause neuroinflammation after tongue instillation. Furthermore, exposure to ZnO NPs led to a reduction in cell viability and induction of inflammatory response and calcium influx in BV2 and PC12 cells. The mechanism underlying how ZnO NPs induce neuroinflammation via the Ca^2+^-dependent NF-κB, ERK and p38 activation pathways was verified at the cytological level.

**Conclusion:**

This study provided a new way how NPs, such as ZnO NPs, induce neuroinflammation via the taste nerve translocation pathway, a new mechanism for ZnO NPs-induced neuroinflammation and a new direction for nanomaterial toxicity analysis.

**Electronic supplementary material:**

The online version of this article (10.1186/s12989-018-0274-0) contains supplementary material, which is available to authorized users.

## Background

Over the past few decades, nanotechnology has developed rapidly and emerged as a promising technique for various materials science, biomedical and daily life applications. Zinc oxide nanoparticles (ZnO NPs) are frequently used in dental materials, such as toothpaste [1], mouthwash, dental resin, root canal flings [2] and implant surface coatings [3]. The increased biological applications of ZnO NPs in stomatology provides a potential route for ZnO NPs to translocate into the human body. Despite great progress in nanotechnology, the effects of ZnO NPs exposure on the human body, in general and specifically on the brain, are not well understood. The central nervous system (CNS), which plays a commander-like role in our body, integrates received information to coordinate and influence all other bodily activities.

The routes of ZnO NPs transfer to the CNS include blood brain barrier(BBB) pathway [4], placental barrier pathway [5, 6], gastrointestinal absorption pathway [7, 8] and sensory nerve translocation pathway [9, 10]. At present, large numbers of studies have shown that intranasal instillation of nanoparticles can be transported along the olfactory nerve and trigeminal nerve into CNS, resulting in neurotoxicity [11–14]. The taste nerve parthway is similar to the olfactory nerve parthway, which can identify the characteristics of different foods and transmit sensory signals to CNS. Therefore, nanoparticles tongue instillation is likely to be uptaken by taste bud cells and transported into CNS via the taste nerve pathway. In a previous study, we demonstrated that ZnO NPs and titanium dioxide NPs (TiO_2_ NPs) can be taken up by taste buds and translocated into the brain via taste nerves (chorda tympani (CT) and glossopharyngeal nerves), providing a new pathway for NPs to translocate into the brain [15]. Furthermore, NPs deposited onto the brain induce oxidative damage and degenerate learning and memory, resulting in neurodegeneration. By definition, neurodegeneration disturbs the properties of the CNS and therefore affects neuronal function, which is associated with many neurodegenerative diseases, such as Alzheimer’s disease (AD), frontotemporal lobar dementia (FTLD), Parkinson’s disease (PD), and amyotrophic lateral sclerosis (ALS). Increasing evidence shows that neurodegenerative diseases are partially caused and promoted by neuroinflammation. Furthermore, neuropathologists have found that glial cells, including astrocytes and microglia, are activated in each of these disorders, accompanied by low-to-moderate levels of inflammatory mediators in the parenchyma [16]. NPs may accelerate the process of neurodevelopmental disorders and neurodegenerative diseases by promoting inflammation, reactive oxygen species (ROS), microglial activation and neuronal loss [17]. Many studies have indicated that exposure to ZnO NPs can induce significant inflammatory responses in various organs, such as lung [18–20], heart [21] and brain. Tian [22] reported that ZnO NPs induced inflammatory responses in the brains of mice and further impaired their learning and memory abilities. Thus, the neurodegeneration caused by tongue-instilled ZnO NPs and TiO_2_ NPs may be associated with neuroinflammation. Therefore, this study aimed to investigate whether tongue-instilled ZnO NPs induce inflammatory responses in the CNS and to evaluate the potential molecular mechanisms underlying this process.

## Results

### Characterization, identification, and stability of ZnO NPs

**Fig. 1:**
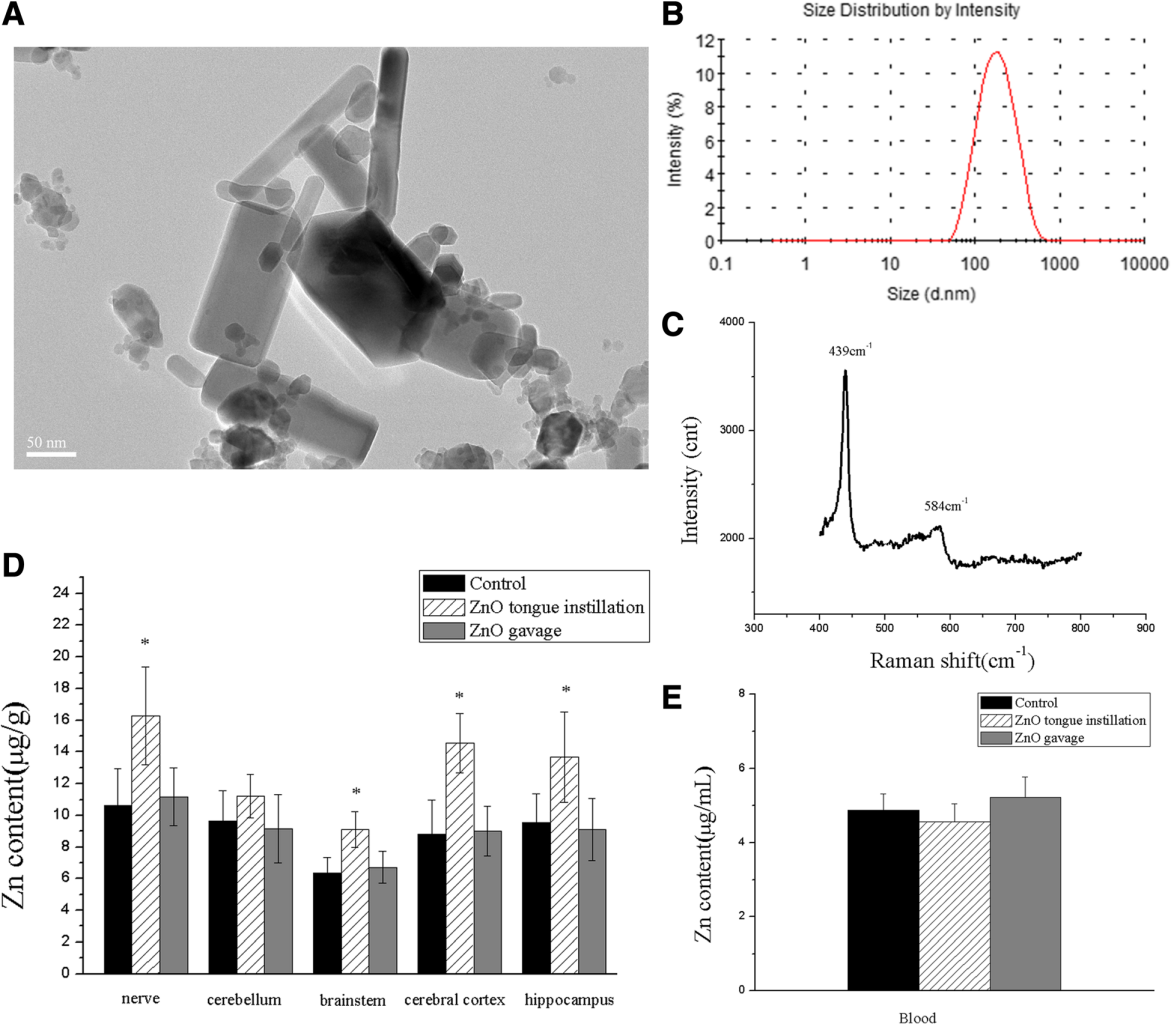
Characterization and biodistribution of ZnO NPs. (**a**) TEM image of morphology and size of ZnO NPs; (**b**) DLS was used to determine ZnO NPs distribution in distilled water; (**c**) Raman spectra of ZnO NPs; The concentrations of Zn in the nerves (CT and glossopharyngeal nerve), sub-brain regions (**d**) and blood (**e**). The Zn levels in the tongue instillation group were significantly higher than those in the ZnO gavage and control groups, while there were no significant different in the blood Zn concentrations in three groups. Results shown as means ± SD from three independent experiments, compare with control group, *P < 0.05. (*n* = 6). Abbreviations: TEM: transmission electron microscopy; ZnO: zinc oxide nanoparticles; CT: chorda tympani; SD: Standard deviation

The physical and primary particle sizes and morphology of ZnO NPs were measured by TEM. The TEM micrographs demonstrated that ZnO NPs were prismatic-shaped, with a diameter of 42.31 ± 17.94 nm (Fig. 1a). The hydrodynamic size of ZnO NPs in a DW suspension was 232.8 ± 53.55 nm, and the size distribution was centralized (Fig. 1b). The Raman spectra of ZnO NPs samples showed peaks at 437 and 584 cm^− 1^, which confirmed the molecular structure of ZnO NPs (Fig. 1c). Furthermore, the zeta potential of ZnO NPs was 14.6 mV at pH 7.0, and the specific surface area was 27.342 (m^2^/g). The physicochemical properties of ZnO NPs are summarized in Table 1.

**Table 1:** Summary of physicochemical properties of ZnO nanoparticles

Particles	Morphology	Primary size (nm)^a^	Hydrodynamic size (nm)^b^	Zeta potential (mV)^b^	Surface area (m^2^/g)
ZnO	Hexagonal	42.31 ± 17.94	232.8	14.6	27.342

^a^The primary nanoparticle size was measured by transmission electron microscope

^b^Nanoparticles were dispersed in DW and measured by dynamic light scattering instrument

### Biodistribution of ZnO NPs

To assess ZnO NPs penetration in rat tissues, the Zn concentrations in nerve and brain tissue as well as those in blood were quantitatively analysed by ICP-MS. The Zn levels in the tongue instillation group were significantly higher than those in the gavage and control groups, and the Zn levels in the sub-brain regions were ranked as follows: cerebral cortex > hippocampus > cerebellum > brain stem pattern (F(2, 51) in nerve, cerebellum, brain stem, cerebral cortex, hippocampus were 3.482, 1.475, 3.672, 3.893, 3.315, respectively) (Fig. 1d). However, in the tongue instillation and gavage groups, there were no significant increases in the blood Zn concentrations compared with that in the control group (F(2, 50) = 1.351) (Fig. 1e). These results confirmed that most of ZnO NPs enter the rat brain via the taste nerve pathway but not via blood and the digestive tract when administered via the tongue.

### Neuroinflammation is induced by ZnO NPs in the rat brain

**Fig. 2:**
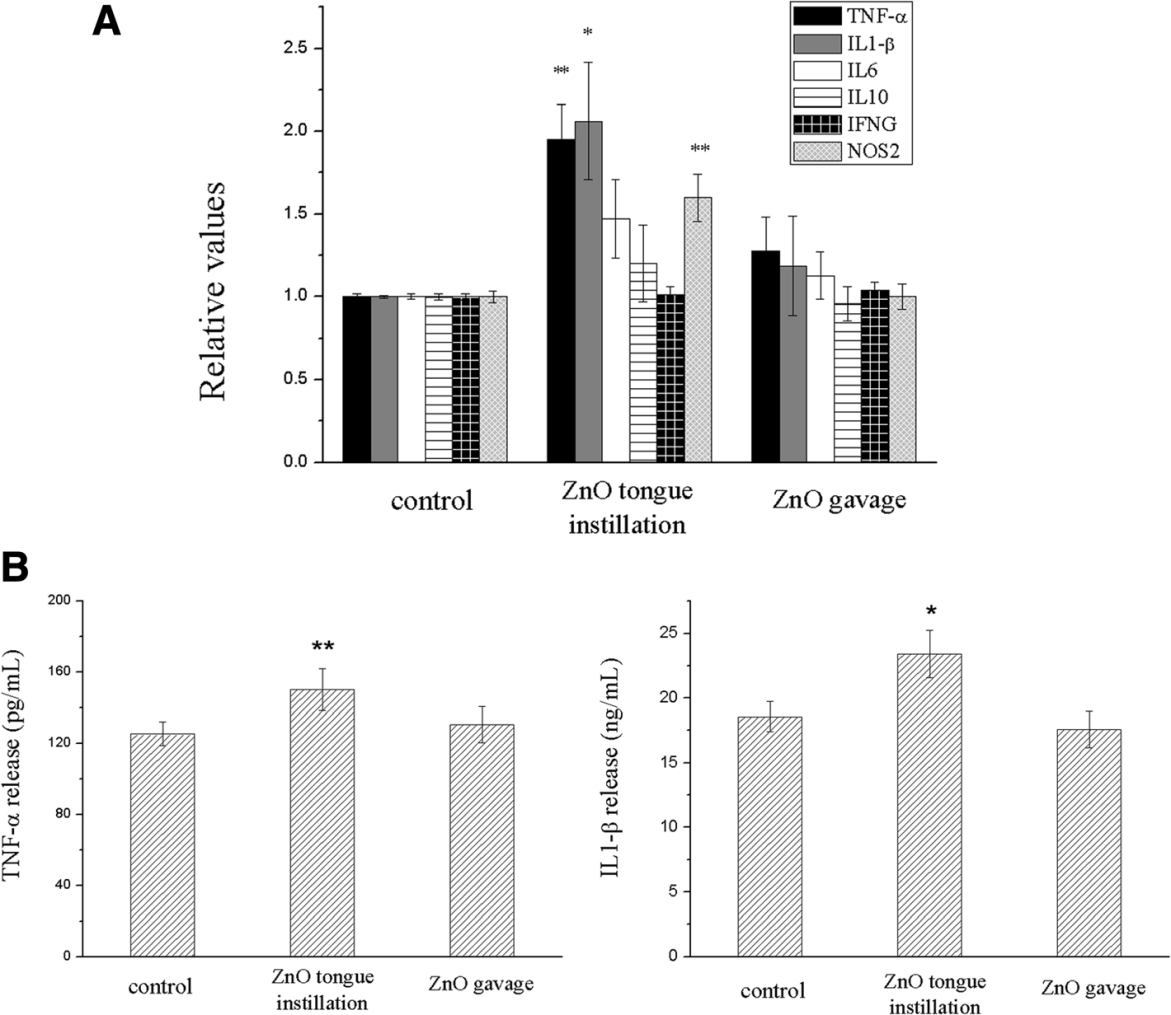
Neuroinflammation is induced by ZnO NPs in the rat brain. (**a**) mRNA relative values of cytokines gene expressions in three groups. (**b**) The concentration of TNF-α and IL-1β were analyzed after 30 days tongue instillation with DW, ZnO NPs and 30 days gavage with ZnO NPs. TNF-α, IL-1β and NOS2 were significantly upregulated in the tongue instillation group compared to that in the other groups. Furthermore, significantly high levels of TNF-α and IL-1β in the brain were detectived in ZnO tongue instillation groups. Results shown as means ± SD from three independent experiments, compare with control group, *P < 0.05, **P < 0.01. (*n* = 6). Abbreviations: ZnO: zinc oxide nanoparticles; DW: distill water; SD: Standard deviation

To evaluate the inflammatory responses induced by ZnO NPs, the relative gene expression levels of key cytokines, including TNF-α, IL-1β, IL-6, IL-10, IFNG and NOS2, in the cerebral cortex were measured after the exposure. TNF-α, IL-1β and NOS2 were significantly upregulated in the tongue instillation group compared to that in the other groups (F(2, 51) = 5.476, 3.855, 5.767) (Fig. 2a). Furthermore, the TNF-α and IL-1β levels were measured by ELISA, revealing significantly high TNF-α (F(2, 48) = 5.372) and IL-1β (F(2, 50) = 3.985) levels induced by ZnO NPs tongue instillation for 30 days (Fig. 2b).

In addition, to exclude the effect of influence factors (ie, anesthesia procedure) on the experimental results, a complementary study was performed. The animals were divided into five groups of six animals each, including the DW instillation group, the ZnO NPs tongue instillation group, the ZnO NPs gavage group, the DW gavage group and the background group (the animals don’t receive any treatment). The analyzed results in the DW gavage group and the background group were no different with DW instilled group, which indicated the neuroinflammation was induced by ZnO NPs tongue instillation and the other treatment in our study (ie, anesthesia procedure) would not affect our results (Additional file 1: Figure S5).

### ZnO NPs-induced histopathological and immunohistochemical changes

**Fig. 3:**
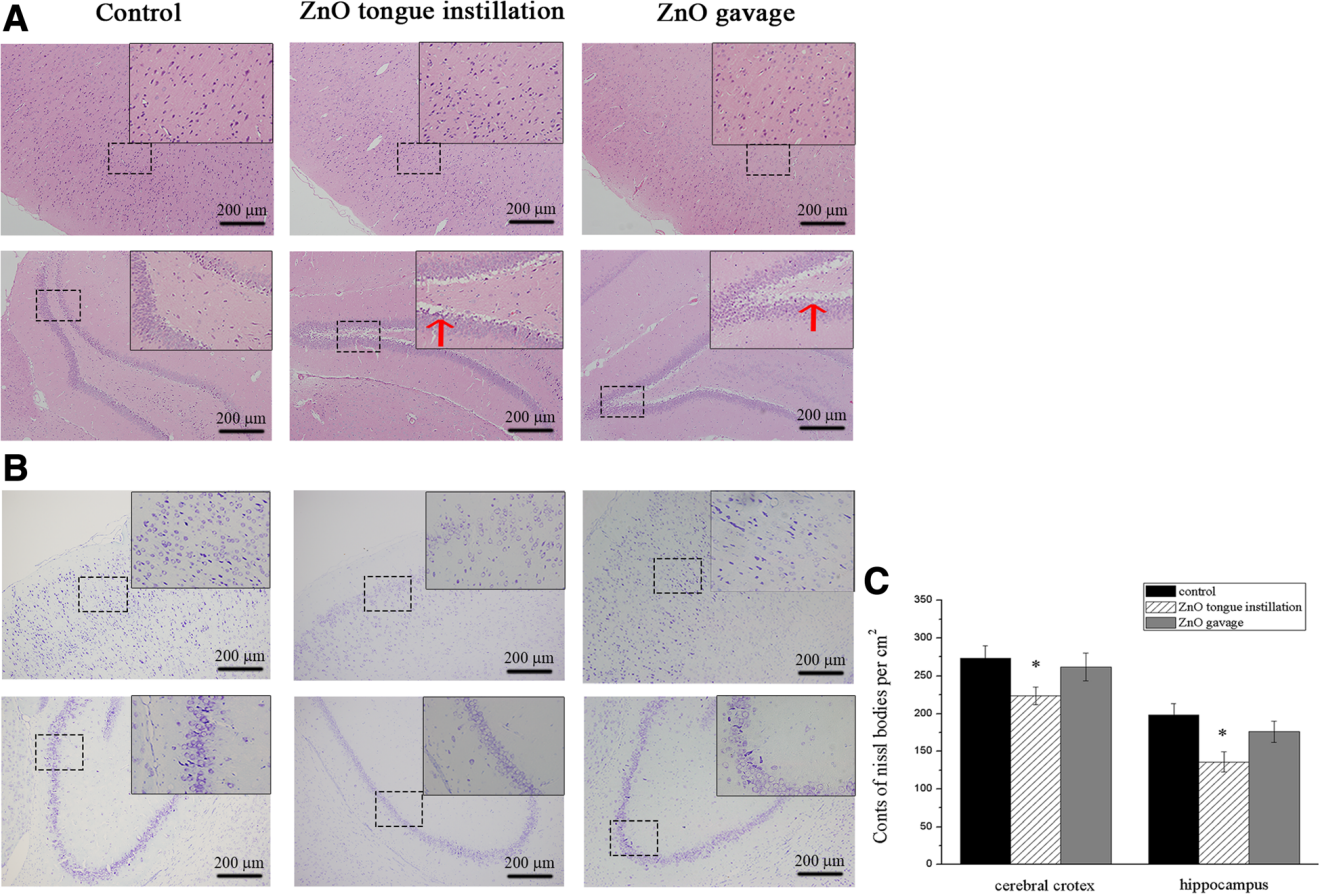
The HE and nissl stains evaluation of brain tissues in rats. Sections of brain tissues with HE (**a**) and nissl stain (**b**). Cell counting analysis of nissl bodies in the cerebral cortex and hippocampus (**c**, number/cm^2^). HE stains showed that there were no apparent changes in brain histology in three groups. However, sparser hippocampal tissues were observed in both ZnO-treated groups compared to that in the control. The red arrows show the sparser hippocampal tissues. Decreased numbers of nissl bodies and shrunken nuclei were found in the cortex and hippocampus in the ZnO NPs tongue instillation group. Results shown as means ± SD from three independent experiments, compare with control group, *P < 0.05, **P < 0.01. (*n* = 5). Abbreviations: ZnO: zinc oxide nanoparticles; HE: hematoxylin & eosin; SD: Standard deviation

**Fig. 4:**
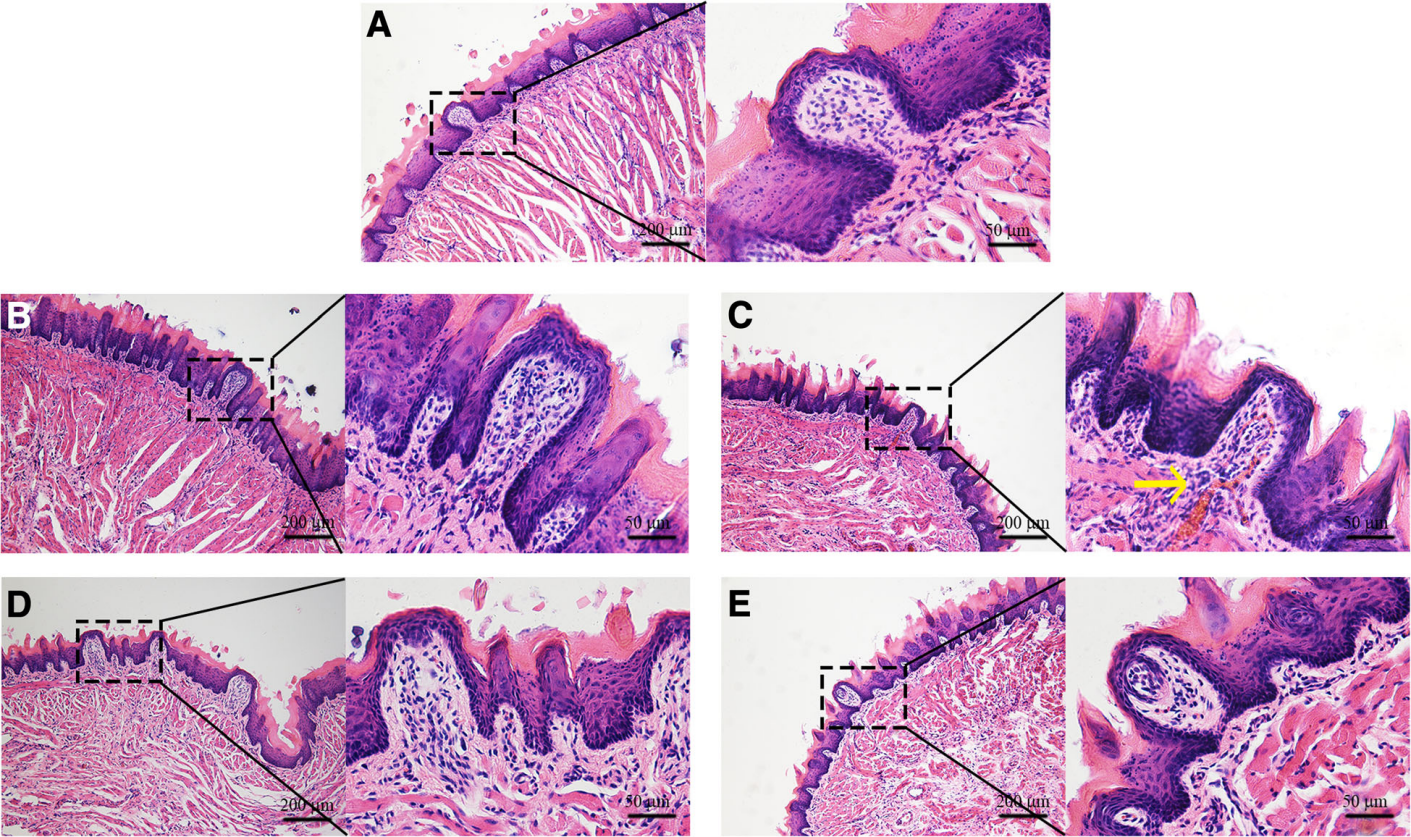
The HE stains evaluation of the lingues in rats. **a** background group; **b** DW instillation group; **c** ZnO NPs tongue instillation group; **d** DW gavage group; **e** ZnO NPs gavage group. There were no apparent changes in lingual structures in each group, while fungiform papilla hyperemia was observed in ZnO tongue instillation group. The yellow arrow showed fungiform papilla hyperemia. (*n* = 6). Abbreviations: ZnO: zinc oxide nanoparticles; HE: hematoxylin & eosin; DW: distilled water

Histopathological analyses of the cerebral cortex revealed that tongue instillation exposure to ZnO NPs induced no apparent changes in brain histology when compared with the gavage and control groups. However, sparser hippocampal tissues were observed in both ZnO-treated groups compared to that in the control (Fig. 3a). Thus, ZnO NPs in the brain did not affect the cellular integrity or tissue morphology but still caused some minor damage. What’s more, fungiform papilla hyperemia was observed in ZnO tongue instillation group (Fig. 4), which may hint the sensory and taste function of tongue was affected.

Pathological changes in brain neurons were also examined using the Nissl staining method. Decreased numbers of Nissl bodies and shrunken nuclei were found in the cortex (F(2, 42) = 3.751) and hippocampus (F(2, 42) = 4.133) after ZnO NPs tongue instillation, which showed that neurons were damaged in this group (Fig. 3b).

**Fig. 5:**
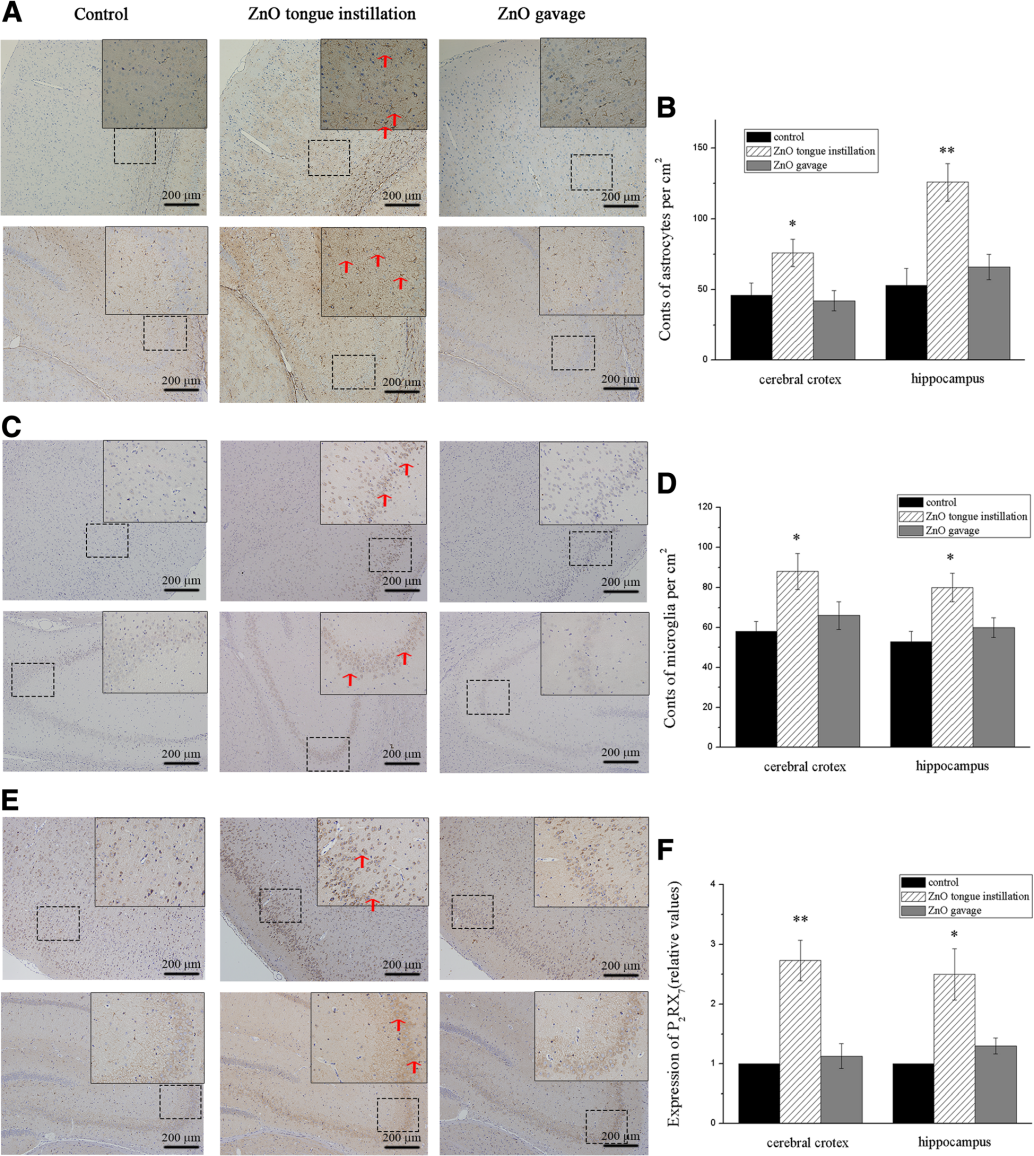
Immunohistochemical analysis of brain tissues in rats. Sections of brain tissues were stained with GFAP (**a**), CD11b (**c**) and P_2_RX_7_ (**e**). Cell counting analysis of astrocytes and microglia in the cerebral cortex and hippocampus (**b** & **d**, number/cm^2^). The relative values of P_2_RX_7_ expression in the cerebral cortex and hippocampus (**f**). GFAP, CD11b and P2X7 receptor expression was increased in the tongue instillation group compared to that in the other groups. The red arrows show the positive cells. Results shown as means ± SD from three independent experiments, compare with control group, *P < 0.05, **P < 0.01. (*n* = 5). Abbreviations: ZnO: zinc oxide nanoparticles; GFAP: glial fibrillary acidic protein; P_2_RX_7_: P_2_X purinoceptor 7; SD: Standard deviation

Immunostaining of the cortex and hippocampus with anti-GFAP and anti-CD11b indicated that GFAP (F(2, 42) = 3.698) and CD11b (F(2, 42) = 4.572) expression was increased in the tongue instillation group compared to that in the other groups, suggesting that ZnO NPs stimulate astrocyte and microglial activation or proliferation after translocation to the brain (Fig. 5a and b). P_2_X_7_ receptor expression was also increased in the ZnO NPs tongue instillation group (F(2, 42) = 6.824), which might be associated with inflammatory responses in the brain (Fig. 5c).

### Dose-dependent and time-dependent cytotoxicity induced by ZnO NPs

**Fig. 6:**
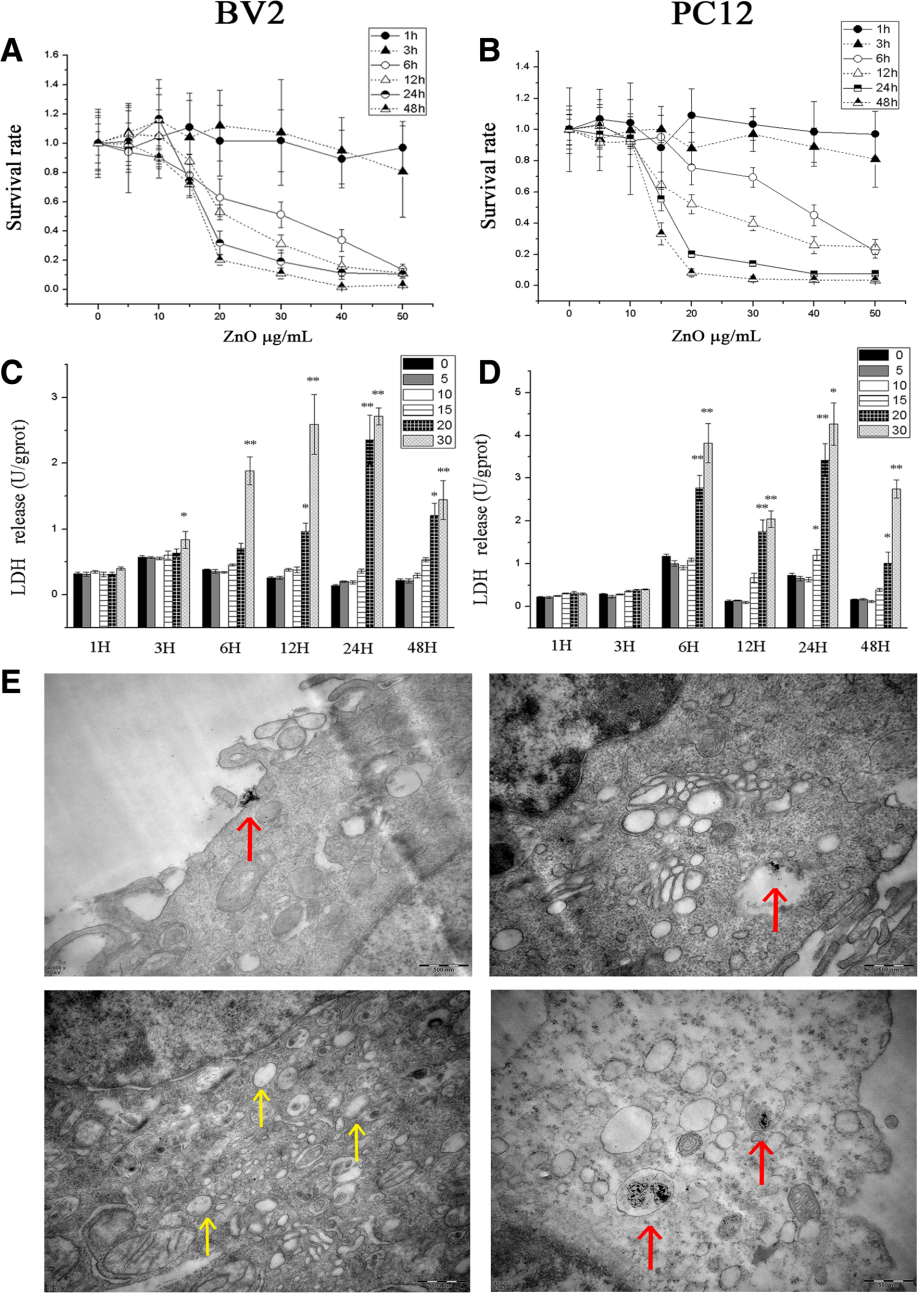
ZnO NPs induced alterations in cells viability and were taken up by BV2 and PC12 cells. BV2 (**a**) and PC12 (**b**) cells were treated with ZnO NPs at doses of 0, 5, 10, 15, 20, 30, 40 or 50 μg/mL for 1, 3, 6, 12, 24 or 48 h. Cell viability was determined using the MTT assay. The LDH level in cell culture medium of BV2 (**c**) and PC12 (**d**) cells. (**e**)TEM images of BV2 and PC12 cells exposed to ZnO NPs (30 μg/mL) for 1 h. ZnO NPs were taken up by PC12 cells and located in the vesicles. What’s more, a sunken with ZnO NPs in the cytomembrane was observed in BV2 cells. However, numerous vacuoles but not granular NPs were observed in the cytoplasm of BV2 cells. The red arrows show the internalization and localization of ZnO NPs. The yellow arrows show the empty vesicles. Results shown as means ± SD from three independent experiments, compare with control group, *P < 0.05, **P < 0.01. Abbreviations: ZnO: zinc oxide nanoparticles; MTT: 3-(4,5-Dimethylthiazol-2-yl)-2,5-diphenyltetrazolium bromide; LDH: lactic dehydrogenase; TEM: transmission electron microscopy; SD: Standard deviation

To elucidate the mechanism of brain inflammation, BV2 and PC12 cells were selected as models for analysis at the cellular and molecular levels. To investigate the cytotoxic effects of ZnO NPs, the viabilities of BV2 and PC12 cells were determined by the MTT and LDH assays. Within 3 h of treatment, ZnO NPs exerted no strong toxic effects on BV2 cells or PC12 cells. Moreover, no significant viability changes were observed in either cell line at concentrations below 10 μg/mL at any time point. However, when the concentrations reached 20 μg/mL, an obvious decrease in cell viability was observed after 6 h of treatment. Henceforth,cell survival rates decrease as time and ZnO NPs concentrations increase. Furthermore, BV2 cells showed more sensitivity to ZnO NPs treatment, as their viability decreased earlier and the survival rate was lower than those in PC12 cells (Fig. 6a and b).

The LDH assay reflects membrane integrity, which can indirectly assess cell viability. As shown in Fig. 6c and d, the LDH activity was increased by ZnO NPs in a concentration- and time-dependent manner. The largest increase was observed at concentrations of 30 μg/mL at every time point (*P* < 0.05) except for 1 h. Dose- and time-dependent reductions in BV2 and PC12 cell viability were observed after exposure to ZnO NPs.

### ZnO NPs were taken up by BV2 and PC12 cells

To evaluate ZnO NPs distribution and the induction of cellular ultrastructural changes, BV2 and PC12 cells were incubated with 30 μg/mL ZnO NPs for 1 h. TEM analysis revealed that ZnO NPs were taken up by PC12 cells and located in the vesicles. What’s more, a sunken with ZnO NPs in the cytomembrane was observed in BV2 cells, which showed the cell was phagocytosing NPs. However, numerous vacuoles but not granular NPs were obseved in the cytoplasm of BV2 cells (Fig. 6e).

### ZnO NPs-induced proinflammatory gene alterations and cytokine release

**Fig. 7:**
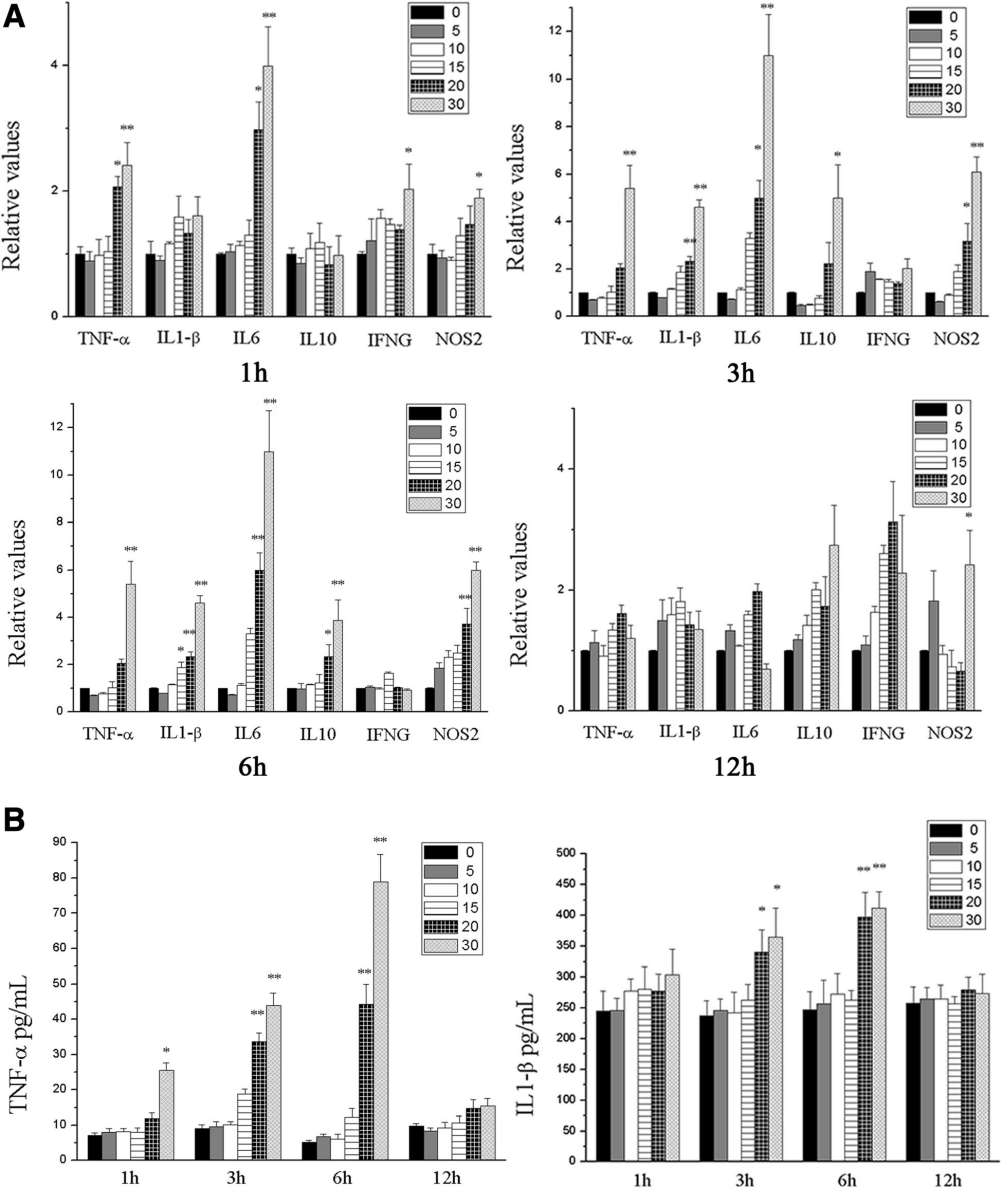
The inflammatory responses to ZnO NPs in BV2 cells. The mRNA relative values of cytokines gene expressions in BV2 cells (**a**). The gene expression levels of TNF-α, IL-1β, IL-6 and NOS2 were significant up-regulation after ZnO NPs exposure at 1, 3, and 6 h, and IL-10 gene expression was significantly increased at 3 and 6 h. The concentration of TNF-α and IL-1β in BV2 cells culture supernatants were performed by Elisa analysis (**b**). The secretion of both cytokines was significantly increased after ZnO NPs stimulation in BV2 cells at a dose ≥20 μg/mL, peaking at 3 and 6 h. Results shown as means ± SD from three independent experiments, compare with control group, *P < 0.05, **P < 0.01. Abbreviations: ZnO: zinc oxide nanoparticles; SD: Standard deviation

To clarify the inflammatory responses to ZnO NPs in BV2 and PC12 cells, the levels of the proinflammatory genes TNF-α, IL-1β, IL-6, IL-10, IFNG and NOS2 were measured by qRT-PCR. After ZnO NPs exposure, significantly increased gene expression levels of TNF-α, IL-1β, IL-6 and NOS2 were observed at 1, 3, and 6 h (*P* < 0.05), and a significant up-regulation of IL-10 gene expression was observed at 3 and 6 h (*P* < 0.05) after 30 μg/mL ZnO NPs treatment in BV2 cells (Fig. 7a). PC12 cells treated with 30 μg/mL ZnO NPs also exhibited increased TNF-α, IL-6 and NOS2 gene expression at 1, 3 and 6 h (*P* < 0.05), but no proinflammatory genes were significantly altered at 12 h (Additional file 2: Figure S1A).

Furthermore, TNF-α and IL-1β in the cellular supernatants were measured. The secretion of both cytokines was significantly increased (*P* < 0.05) after ZnO NPs stimulation in BV2 cells at a dose≥20 μg/mL, peaking at 3 and 6 h (Fig. 7b). In addition, ZnO NPs also induced the secretion of TNF-α in PC12 cells (*P* < 0.05) (Additional file 2: Figure S1B), while the cytokine release dropped after 12 h of exposure in both cell lines. These data demonstrated that ZnO NPs dose-dependently induce expression cytokine and proinflammatory gene expression, especially after 6 h of exposure.

### ZnO NPs increase intracellular Ca^2+^ concentrations

**Fig. 8:**
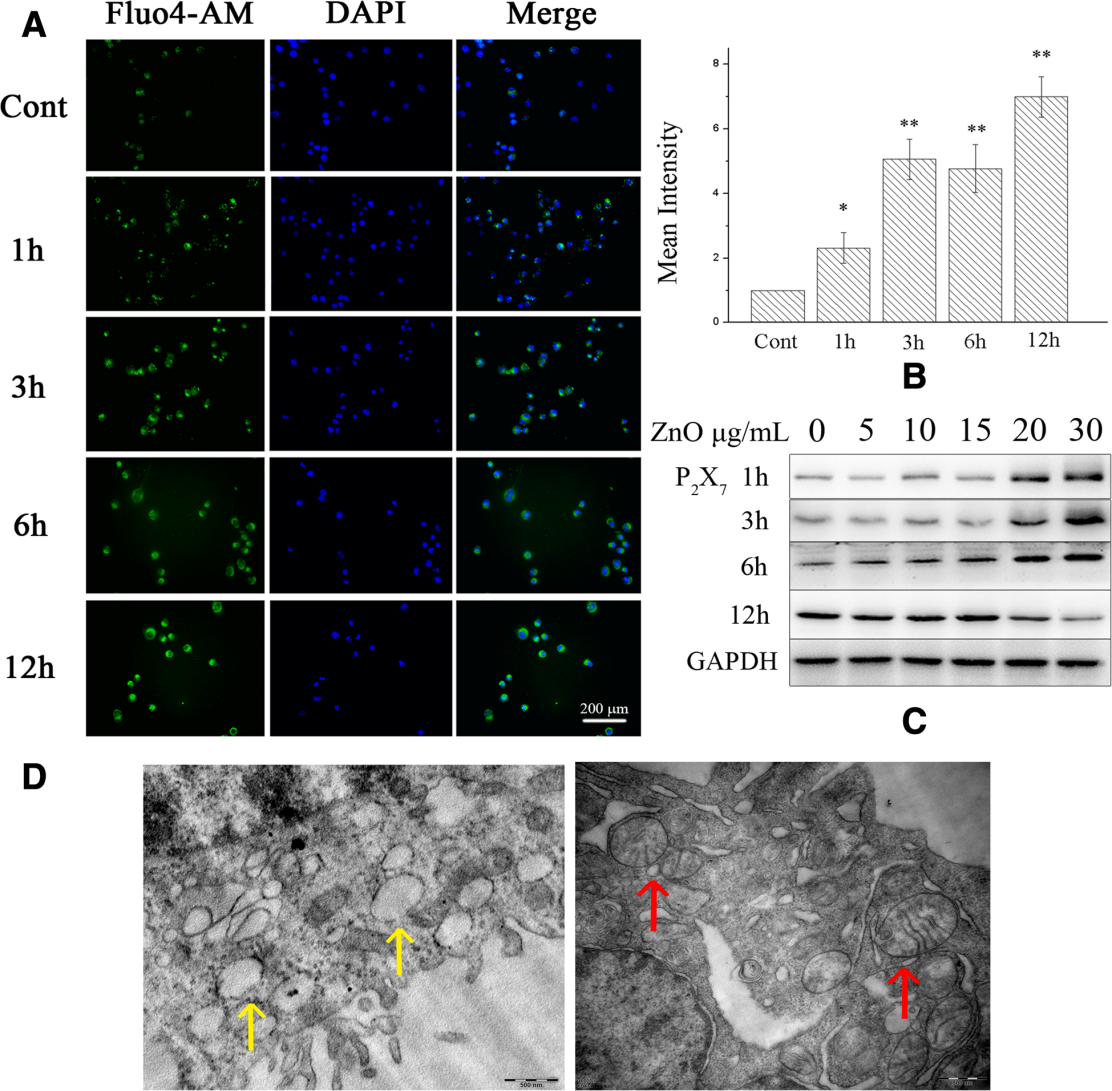
Effect of ZnO NPs on the intracellular Ca^2+^, expression of P_2_X_7_ receptor and ultrastructure changes in BV2 cells. (**a**) BV2 cells were treated with ZnO NPs (30 μg/mL) for 1, 3, 6 or 12 h, cells were stained with Ca^2+^ indicator Fluo4-AM (green) and DAPI (blue). Scale bar represents 200 μm. Compare the mean fluorescence intensity of Ca^2+^ measured in BV2 cells (**b**), and an obvious increase in the Ca^2+^ fluorescence(green) was observed in a time-dependent manner. The expression of P_2_X_7_ receptor was increased in a dose-dependent manner after treating with ZnO NPs in BV2 cells at doses of 0, 5, 10, 15, 20 or 30 μg/mL for 1, 3, 6 or 12 h (**c**). The dilatational endoplasmic reticulum and the empty and swollen mitochondria were observed after ZnO NPs (30 μg/mL) for 6 h treatment in BV2 cells (**d**). The yellow arrows show the dilatational endoplasmic reticulum and the red arrows show the swollen mitochondria. Results shown as means ± SD from three independent experiments, compare with control group, *P < 0.05, **P < 0.01. Abbreviations: cont: control; ZnO: zinc oxide nanoparticles; P_2_X_7_: P_2_X purinoceptor 7; DAPI: 4,6-diamino-2-phenyl indole; SD: Standard deviation

We detected intracellular Ca^2+^ levels after exposure to 30 μg/mL ZnO NPs, revealing that calcium ions were detected rapidly (after 1 h) in BV2 (F(4, 55) = 3.571) and PC12 cells (F(4, 55) = 3.079) (Fig. 8a and Additional file 3: Figure S2A).

P_2_X_7_, an ionotropic receptor, was detected by western blot to elucidate the factors underlying the intracellular Ca^2+^ increase. P_2_X_7_ expression was increased in a dose-dependent manner in both cell lines at 1, 3, and 6 h after ZnO NPs treatment. However, P_2_X_7_ expression was drastically reduced at 12 h, which might be caused by the low cell viability observed at 12 h (Fig. 8c and Additional file 3: Figure S2C).

Furthermore, cell ultrastructural changes indicated that the calcium stores (mitochondria and endoplasmic reticulum) were injured after treatment with 30 μg/mL ZnO NPs. The dilatational endoplasmic reticulum and the empty and swollen mitochondria were observed in the cytoplasm (Fig. 8d and Additional file 3: Figure S2D).

### Mechanism underlying ZnO NPs-induced inflammation

**Fig. 9:**
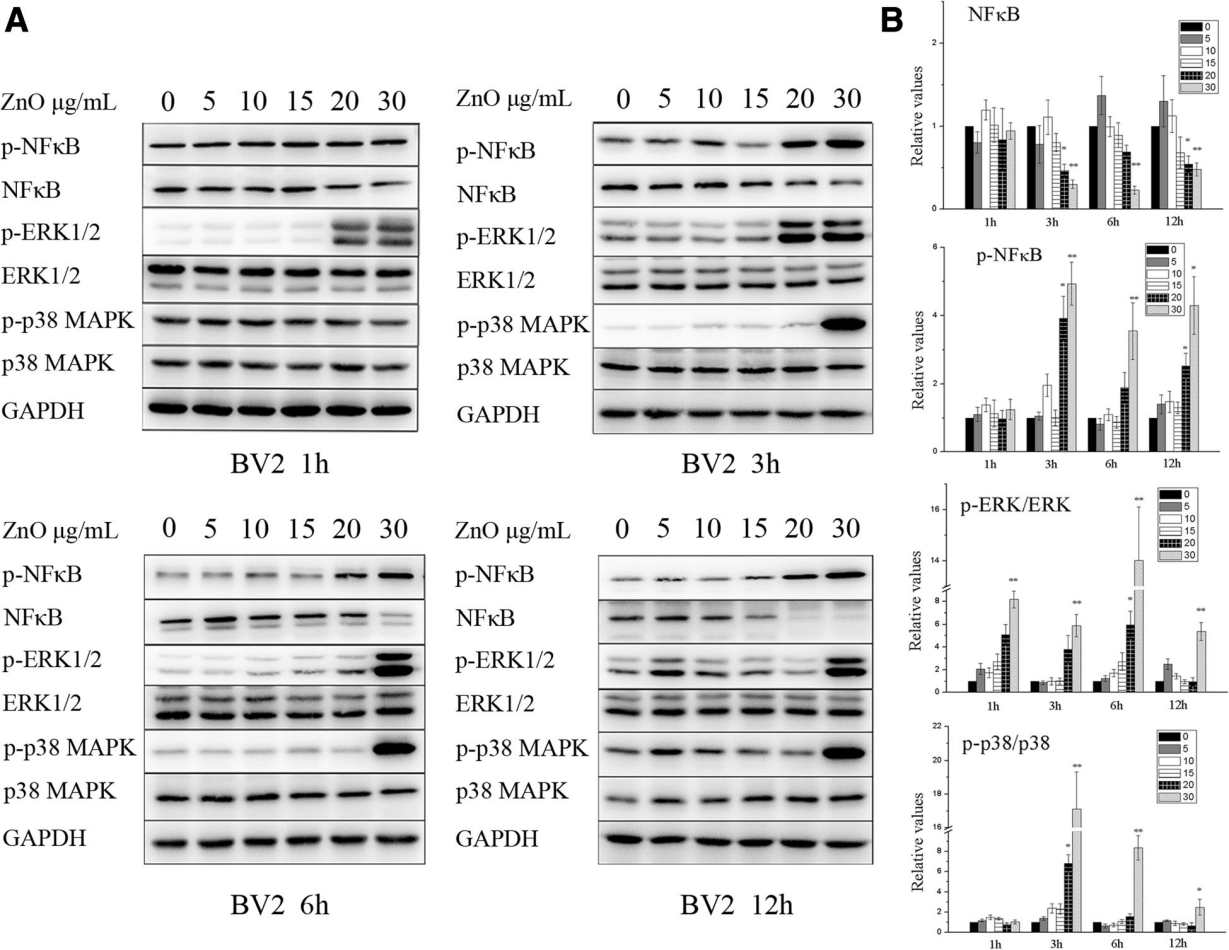
The expression of involved mechanism of inflammation after treatment with ZnO NPs. BV2 cells were treated with ZnO NPs at a dose of 0, 5, 10, 15, 20 or 30 μg/mL for 1, 3, 6 or 12 h. Total proteins were extracted, and the levels of NF-κB, ERK and p38 signaling pathway molecules were analyzed via Western Blot (**a**). The gray value was semiquantitative as shown in histogram (**b**). The phosphorylation levels of NF-κB, ERK and p38 were significantly increase after ZnO NPs treatment. Results shown as means ± SD from three independent experiments, compare with control group, *P < 0.05, **P < 0.01. Abbreviations: ZnO: zinc oxide nanoparticles; SD: Standard deviation

To elucidate the possible molecular mechanisms involved in ZnO NPs-induced toxicity, western blot analysis was used to evaluate the protein expression of NF-κB, ERK and p38. As shown in Fig. 9a, the phosphorylation of ERK in BV2 cells was initially increased after incubation with 30 μg/mL ZnO NPs for 1 h (F(5, 48) = 3.079). Meanwhile, significantly increased phosphorylation levels of NF-κB, ERK and p38 were observed in both cell lines after 30 μg/mL ZnO treatment beginning at 3 h and continuing through 12 h (*P* < 0.05) (Fig. 9 and Additional file 4: Figure S3). Furthermore, total NF-κB in the cytoplasm was decreased, accompanied by increased NF-κB phosphorylation in the cytoplasm, suggesting that NF-κB p65 translocates into the nucleus and leads to NF-κB activation (Fig. 9 and Additional file 4: Figure S3). These analyses suggest that the NF-κB, ERK and p38 pathways participate in ZnO NPs-induced inflammation in a dose-dependent manner.

### Intracellular Ca^2+^ plays an important role in ZnO NPs-induced inflammation

**Fig. 10:**
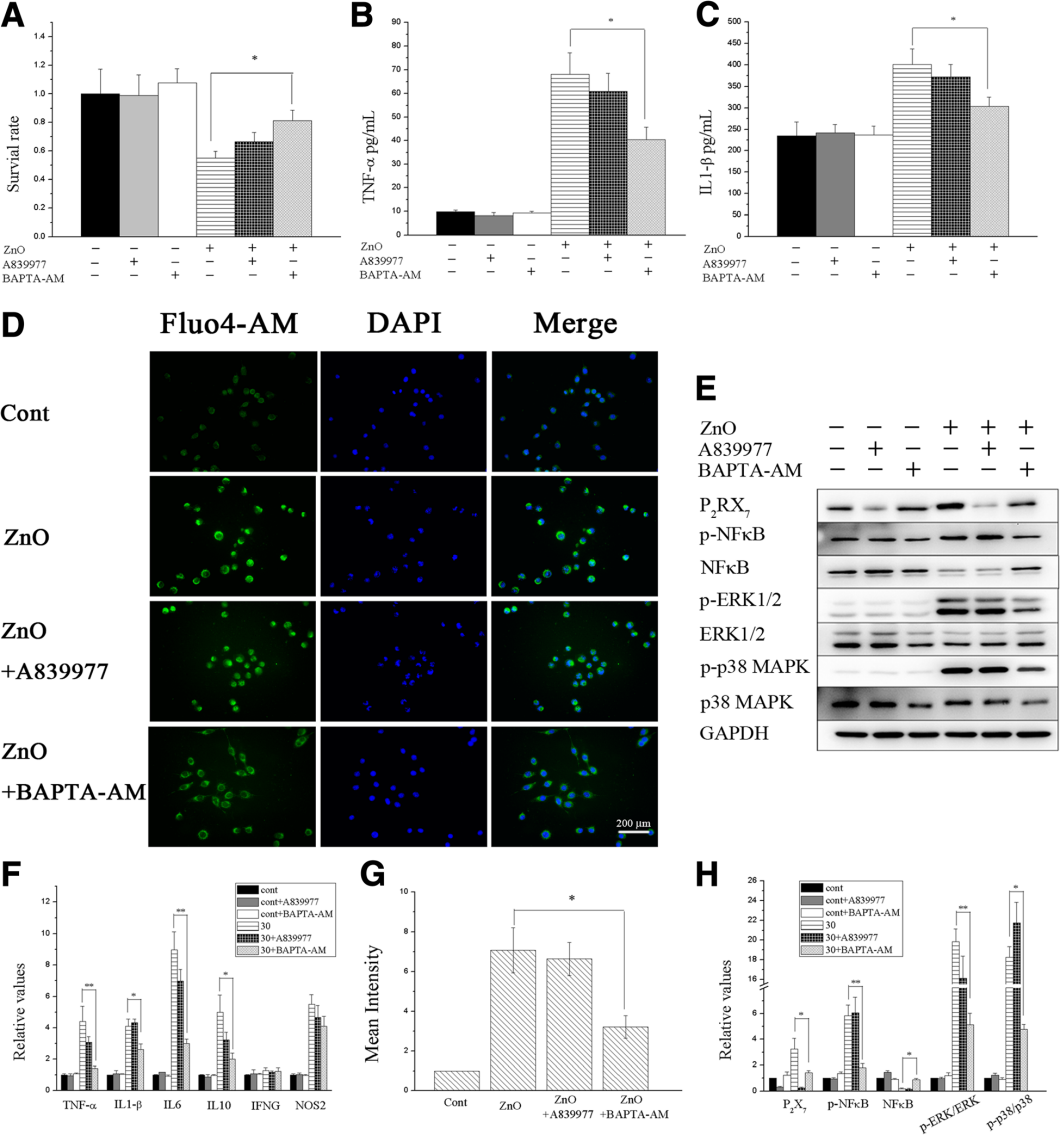
ZnO NPs-induced inflammation is mediated by calcium-dependent pathways in BV2 cells. BV2 cells were preincubated for 1 h with A839977 (200 nM) and BAPTA-AM (20 μM) before ZnO NPs treatment. After the cells were treated with ZnO NPs at a dose of 30 μg/ml for 6 h. Summary of cell viability (**a**), TNF-α (**b**) and IL-1β (**c**) release and the expression of proinflammatory genes (**f**). The concentration of Ca^2+^ in BV2 cells, cells were stained with Ca^2+^ indicator Fluo4-AM (green) and DAPI (blue) (**d**). Scale bar represents 200 μm. Compare the mean fluorescence intensity of Ca^2+^ measured in BV2 cells (**g**). Total proteins of BV2 cells were extracted, and the levels of P_2_X_7_, NF-κB, ERK and p38 signaling pathway molecules were analyzed via Western Blot (**e**). The gray value was semiquantitative as shown in histogram (**h**). The 30 μg/mL ZnO NPs-induced cytotoxicity, increase in proinflammatory cytokine release and NF-κB, ERK and p38 phosphorylation was significantly inhibited by BAPTA-AM. Results shown as means ± SD from three independent experiments, compare with control group, *P < 0.05, **P < 0.01. Abbreviations: cont: control; ZnO: zinc oxide nanoparticles; P_2_X_7_: P_2_X purinoceptor 7; DAPI: 4,6-diamino-2-phenyl indole SD: Standard deviation

To test the possibility that ZnO NPs-induced inflammation is mediated by calcium-dependent pathways, the cells were treated with A839977 (a P_2_X_7_ receptor antagonist) and BAPTA-AM (an intracellular Ca^2+^ chelator). According to previous experiments, the cytotoxicity, inflammation response and increase in the expression of inflammation-related pathways peaked at 6 h after treatment with 30 μg/mL ZnO NPs; thus, 6 h of 30 μg/mL ZnO NPs treatment was used in the inhibition experiments. As shown in Fig. 10e and Additional file 5: Figure S4E, the 30 μg/mL ZnO NPs-induced increase in NF-κB, ERK and p38 phosphorylation was significantly inhibited by BAPTA-AM in both cell lines (*P* < 0.05). The cytokine release and proinflammatory gene expression levels nearly returned to baseline (Fig. 10b, c and f and Additional file 5: Figure S4B, C and F) following the down-regulation of NF-κB, ERK and p38 phosphorylation, and the survival rates also increased after BAPTA-AM pre-treatment, suggesting that excess intracellular Ca^2+^ activates the NF-κB, ERK and p38 pathways, ultimately leading to inflammatory responses and apoptosis. However, the levels of ZnO NPs-induced cytokine release and proinflammatory gene expression were not significantly decreased after treatment with A839977. Furthermore, in A839977 treatment group, the phosphorylation levels of NF-κB, ERK and p38 were not much difference compare with control group, perhaps due to the failure of intracellular Ca^2+^ levels to return to baseline by blocking P_2_X_7_ receptor (Fig. 10d and Additional file 5: Figure S4D). Together, these results indicate that intracellular Ca^2+^ plays an important role in ZnO NPs-induced neuroinflammation.

## Discussion

The wide applications of NPs have raised concerns regarding their safety when released into the environment and the human body. Previous studies indicated that the toxicological effects of NPs were associated with their size [23–25], shape [26–28], surface area [29], ζ-potential [8, 25] and surface modifications [30–32]. Characterization analysis indicated that ZnO NPs used in our study are small in size and have large surface areas, positive charges and good dispersion, allowing easier cellular uptake than NPs of larger sizes, smaller surface areas and agglomerative clustering. Therefore, ZnO NPs have more surface activity and enhanced adsorption properties, which might have unusual biological effects when compared to other substances that we contact in daily life.

In our previous study, we demonstrated that ZnO NPs and TiO_2_ NPs can transfer into the CNS via taste nerves after tongue instillation for 30 days. Although tongue- instilled ZnO NPs and TiO_2_ NPs didn’t cause detectable influence on rat growth and development, they induced oxidative damage, learning and memory degeneration and neurodegeneration in a Wistar rat model [15]. Based on the complicated relationships between neuroinflammation and neurodegenerative disease [16], we speculated that the neurodegeneration caused by the tongue instillation of ZnO and TiO_2_ NPs may be associated with neuroinflammation. Therefore, this study aimed to investigate whether tongue-instilled ZnO NPs induce inflammatory responses in the CNS and the potential molecular mechanisms underlying this process.

The first step to assessing the neuroinflammation induced by ZnO NPs was to determine their fate in the brain. The tongue instillation of ZnO NPs for 30 days significantly increased the Zn content in the brain, and the Zn levels in the sub-brain regions were ranked as follows: cerebral cortex > hippocampus > cerebellum > brain stem. The concentrations of Zn in liver, spleen, kidney, and brain tissues were reportedly increased substantially after 13 weeks of repeated oral ZnO NPs administration (536.8 mg/kg BW), while no significant differences in Zn concentrations were observed between the vehicle control and ZnO NPs (134.2 mg/kg BW) administered orally [33]. The exposure concentrations of ZnO NPs were more than the 500 mg/kg generally administered via gavage in toxicology studies [6, 34, 35]. Thus, increasing brain Zn concentrations requires a much higher dose of ZnO NPs and iterative administration through gavage treatment than other exposure methods. After receiving a single dose of 2000 mg/kg ZnO NPs by oral gavage, the highest plasma Zn levels were observed at 24 h, but the Zn levels were barely altered in the 50 mg/kg treatment group at this time point [36]. However, in our study, the Zn levels in the tongue instillation group were significantly higher than those in the gavage and control groups in the taste nerve and sub-brain regions, while there was no significant increase in blood Zn levels, indicating that most of ZnO NPs are transferred to the brain via taste nerves but do not enter the digestive tract or blood.

The deposited NPs in the brain would damage neuronal function and induce neuronal injury. Histopathological analyses of the brain revealed that exposure to ZnO NPs via tongue instillation did not affect cellular integrity or tissue morphology. However, the Nissl method, used to highlight important structural features of neurons, indicated that neurons were damaged after ZnO NPs tongue instillation. Wang et al. [37] also found that intranasally instilled TiO_2_ NPs induced pathological changes and abnormal neuronal arrangement in the hippocampus and CNS. Healthy and integrated brain neurons are essential for learning and memory. Dose- and time-dependent reductions in the viabilities of BV2 and PC12 cells were observed after exposure to ZnO NPs. The results from this study demonstrated that ZnO NPs had a severe neural toxicological effect. In addition, fungiform papilla hyperemia was observed in ZnO tongue instillation group. The damage of lingual papilla and neurons may affect the taste sensations, which need to be further study.

The activation of glial cells, including astrocytes and microglia, which are the first line of defence against the entry of foreign particles or infectious agents, can be identified by GFAP and CD11b, respectively. In present study, ZnO NPs stimulate astrocyte and microglial activation or proliferation after translocation to the brain. Overactivated glial cells may induce ROS, release nitric oxide (NO) and inflammatory cytokines, and induce neurotoxicity in response to extraneous nanoparticles [38]. Furthermore, activated astrocytes and microglia are regarded as characteristics of neuroinflammatory diseases [39]. TNF-α and IL-1β release and proinflammatory gene expression alterations in the brain after 30 days of tongue instillation. TNF-α release is always accompanied by glial cell activation, leukocyte infiltration and neuronal apoptosis [40, 41]. IL-1β is central to the inflammatory process as well, attributed to the increased permeability of the BBB to circulating immune cells, and is directly responsible for the decline in tissue function [42]. What’s more, cytotoxicity was examined in accordance with the intracellular levels of inflammation in our vitro study. These findings indicated that inflammatory response might be a key route in the neurotoxicity of ZnO NPs.

BV2 cells showed more sensitive to the treatment of ZnO NPs compared with PC12 cells, as their viability decreased earlier and the survival rate was lower than those in PC12 cells. Meanwhile, ZnO NPs induced severer inflammatory responses in BV2 cells. TEM images showed that ZnO NPs were taken up by PC12 cells and located in the vesicles, while numerous vacuoles but not granular NPs were observed in the cytoplasm of BV2 cells. In our previous study, we determined that the intracellular ZnO NPs initially remained in lysosomes and then released zinc ions as rapidly as possible in BV2 cells [43]. Microglial cells function like macrophages by acting as a first line of defence against foreign particle or infectious agent entry, which have powerful phagocytic ability and lysosomal system after being activated. ZnO NPs release zinc ions as soon as possible once they locate in lysosomes, so that it is hardly to observe the grainy ZnO NPs in BV2 cells. Fukui et al. [44] indicated that cellular uptake of the ZnO NPs could release numerous Zn^2+^, which caused stronger oxidative stress, and the increase of intracellular Zn^2+^ was the main reason to induce toxicity. Compare with PC12 cells, ZnO NPs can be taken up faster and more by BV2 cells, and then release zinc ions to play a role in inducing cytotoxicity, which may be the reason why BV2 cells showed more sensitive to the treatment of ZnO NPs.

In all eukaryotic cells, finely tuned changes in Ca^2+^ modulate a variety of intracellular functions, and disruption of Ca^2+^ handling leads to cell death [45]. Changes in intracellular calcium ion concentrations are closely association with the progression of inflammation. Previous studies found that NPs increased intracellular Ca^2+^ concentrations and further contributed to the inflammatory response [46, 47]. Herein, calcium ion levels were significantly increased after exposure to 30 μg/mL ZnO NPs in both BV2 and PC12 cells, prompting the consideration that Ca^2+^ influx might be a key factor in ZnO NPs-induced neuroinflammation. Ca^2+^ influx is essential for TNF-α and IL-1α release in murine microglial cells (MG5) and murine peritoneal macrophages, respectively [48, 49]. Previous studies have shown that the executive functions of activated microglia, such as phagocytosis [50], as well as the release of NO, TNF-α, IL-6 and IL-12 are Ca^2+^-dependent [51–53].

ZnO NPs induce membrane lipid peroxidation and lead to calcium influx through membrane channels, inducing further cytotoxicity [54]. Ca^2+^ increases can be elicited by both Ca^2+^ release from intracellular stores and Ca^2+^ influx through plasma membrane channels, which can be divided into four classes: (i) voltage-operated Ca^2+^ channels, (ii) ligand-gated Ca^2+^ channels, (iii) second messenger–operated channels, (iv) store-operated Ca^2+^ entry [45]. Among these classes, purinergic receptors are the most noticeably expressed ligand-gated Ca^2+^-permeable channels in glial cells and control several glial functions, such as cytokine release, in Ca^2+^-dependent pathways [51]. Furthermore, purinergic receptors are also involved in neuronal physiology and pathology, causing neurodegenerative diseases [55, 56].

The P_2_X purinoceptor 7 receptor (P_2_RX_7_) stands out as the single member of the P_2_XR family that plays an important role in multiple inflammatory and immune responses via the inward flux of Na^+^ and Ca^2+^ and the outward flux of K^+^, activating inflammasomes and ultimately contributing to inflammation and host defence [57]. Previous studies indicated that P_2_RX_7_ activates glial cells and promotes the release of proinflammatory factors and chemokines, such as IL-6, TNF, and CXCL2 from mouse microglia [58–60]; MCP-1, CCL2, and IL-1β from rat astrocytes [61, 62]; and IL-8 from rat C6 glioma cells [63]. From the findings above, we hypothesized that P_2_RX_7_-mediated Ca^2+^ influx participate in ZnO NPs-induced neuroinflammation. In the ZnO NPs tongue instillation group, a significant increase in P_2_RX_7_ expression in the cerebral cortex and hippocampus. What’s more, we observed increased P_2_X_7_ protein expression accompanied by high Ca^2+^ concentrations in BV2 and PC12 cells. These results confirmed our hypothesis. In addition, the overloading of intracellular Ca^2+^ may destroy Ca^2+^ stores and further induces apoptosis [64]. Mitochondria and endoplasmic reticulum are the Ca^2+^ stores in the cytoplasm, which stockpile more than 90% of Ca^2+^ inside the cells. TEM analysis indicated significantly damaged mitochondria and endoplasmic reticulum after treatment with 30 μg/mL ZnO NPs. In these cells, numerous vacuoles were observed in the cytoplasm, the endoplasmic reticulum was dilatational and the mitochondria were empty and swollen. Such ultrastructural changes were also previously reported upon treatment with anatase TiO_2_ NPs and PEGylated multi-walled carbon nanotubes [65, 66].

To explore the possible molecular mechanisms underlying ZnO-NPs-mediated neuroinflammation, we measured the expression levels of regulators involved in the inflammation pathway, including NF-κB, ERK and p38. NF-κB is a protein complex involved in cellular responses to stimuli, such as stress, cytokines and NPs [67], and results in inflammation, apoptosis, tumorigenesis, and various autoimmune diseases. In addition, ERK and p38 are members of MAPKs, are important for transducing signals to the nucleus and also play key roles in the regulation of many cellular responses, such as inflammation, proliferation, differentiation and apoptosis [68]. Some studies have indicated that NPs, such as ZnO, Al_2_O_3_, TiO_2_, CeO_2_, Ag, CuO and quantum dots, induce inflammation due to NF-κB [69–71], ERK [72, 73] and/or p38 [74–76] activation. In our study, we also observed similar results, as the NF-κB, ERK and p38 pathways were activated in a dose-dependent manner after ZnO treatment in both cell lines. Furthermore, the activation strength and duration of the ERK pathway differed between the two cell lines, with ERK activation occurring very early (at 1 h) in BV2 cells. As indicated previously, BV2 cells act as the first line of defence against the entry of foreign particles or infectious agents, which potentially contributed to the differential activation of inflammation signalling pathways.

Ca^2+^ is known to play a key role in activating several signalling pathways, such as MAPKs and redox-sensitive transcription factors, including NF-κB, which can lead to the production of proinflammatory molecules and mediators. Previous studies indicated that ultrafine particles and TiO_2_ NPs may exert proinflammatory effects by modulating intracellular calcium concentrations, NF-κB activation, and cytokine production via an ROS-mediated mechanism [77, 78]. In another study, Ca^2+^ influx via ROS-activated TRPM2 mediated the amplification of ERK activation and NF-κB nuclear translocation, which led to CXCL8 production [79]. Ratner also found that *P. aeruginosa* PAO1 stimulated increases in Ca^2+^, and the response stimulated p38 and ERK signalling cascades, resulting in NF-κB activation and IL-8 expression [80]. ROS might be a key mediator of inflammatory conditions, potentially stimulated by increased intracellular Ca^2+^, and further activate the NF-κB, ERK and p38 pathways, resulting in inflammation. Our previous studies also found that ZnO NPs induced oxidative damage in the brain after tongue instillation for 30 days, and ROS levels were enhanced in BV2 cells after 10 μg/mL NPs treatment [15, 81]. Furthermore, enhanced Ca^2+^ levels can lead to the activation of protein kinase C (PKC), which is involved in the activation of NF-κB and ERK [82–84]. Thus, we speculated that ZnO NPs induce neuroinflammation via the Ca^2+^-dependent NF-κB, ERK and p38 activation pathways.

**Fig. 11:**
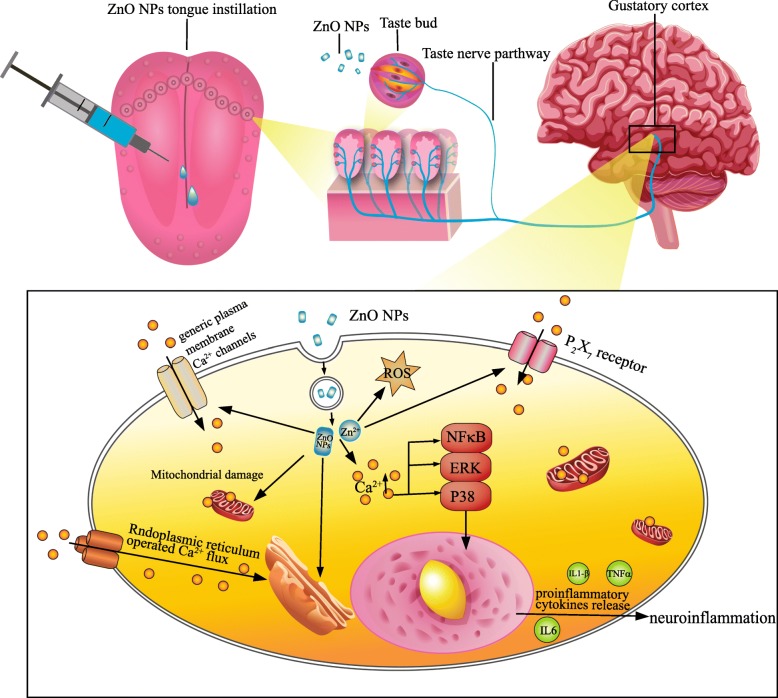
Model of ZnO NPs-induced neuroinflammation in the CNS via the taste nerve pathway and the related mechanisms underlying ZnO NPs-induced neuroinflammation. ZnO NPs could be taken up by taste buds and transfer to CNS via taste nerve pathway, which would further induce neuroinflammation. The cytological experiments show that ZnO NPs get into cells in the CNS (microglia and neuron), which induce LDH and Ca^2+^ release. The increases of Ca^2+^ can be elicited by multiple plasma membrane channels. Subsequently, the increase Ca^2+^ activate NF-κB, ERK and p38 signaling pathway, cause the release of proinflammatory cytokines and neuroinflammation. Abbreviations: ZnO: zinc oxide nanoparticles; CNS: central nervous system

To further elucidate how ZnO NPs-induced inflammation is mediated by calcium-dependent pathways, A839977 and BAPTA-AM were used to block P_2_RX_7_ expression and Ca^2+^ increase. BAPTA-AM but not A839977 prevented the NF-κB, ERK and p38 activation, proinflammatory gene upregulation, TNF-α and IL-1β release and cell viability decrease induced by ZnO NPs. Intracellular Ca^2+^ analysis indicated that BAPTA-AM inhibited Ca^2+^ increases in the cytoplasm, while A839977 did not. As previously mentioned, the P_2_X_7_ receptor is not the only channel to mediate Ca^2+^ influx, as multiple channels participate in this process. ATP-induced biphasic Ca^2+^ mobilization is mediated by P_2_Y receptors (0–5 min), P_2_X_7_ receptors (5–30 min) and internal Ca^2+^ stores (30 min-3 h) [48]. Daniel F. Gilbert et al. [85] found that ATP appears to more selectively induce P_2_X than P_2_Y receptor-operated Ca^2+^ entry and then activates downstream proinflammatory signalling in BV2 cells. During thioglycolate-elicited macrophage activation, PGE2 selectively impairs P_2_Y but not P_2_X_7_ Ca^2+^ mobilization, while this effect is absent in lipopolysaccharide (LPS)-activated cells [86]. Therefore, the relative importance of Ca^2+^ influx versus Ca^2+^ mobilization depends on the stimulus and the cell type. These results indicated that Ca^2+^ increase is essential for ZnO NPs-induced neuroinflammation via the NF-κB, ERK and p38 activation pathways. A model of ZnO NPs-induced neuroinflammation in the CNS via the taste nerve pathway and the related mechanisms underlying ZnO NPs-induced neuroinflammation described herein are shown in Fig. 11.

## Conclusion

In summary, this study demonstrated that ZnO NPs can be transported to the brain via the taste nerve after 30 days of tongue instillation and induce glial cell activation and inflammatory responses in the CNS. Moreover, ZnO NPs can induce inflammatory responses via the Ca^2+^-dependent NF-κB, ERK and p38 activation pathways in BV2 and PC12 cells. In general, this study provided a new way for how NPs, such as ZnO NPs, induce neuroinflammation via the taste nerve translocation pathway (sensory nerves pathway), a new mechanism for ZnO NPs-induced neuroinflammation and a new direction for nanomaterial toxicity analysis. In addition, the findings of this study could provide more useful toxicological information and references for security application of nanomaterials, and some information to prevent and cure neurodegenerative diseases.

## Methods

### Characterization of ZnO NPs

ZnO NP powder was purchased from Sigma­Aldrich (CAS number: 1314-13-2, USA). The physical and primary particle sizes and morphology were determined using transmission electron microscopy (TEM; JEOL, Tokyo, Japan). Raman spectra were acquired at room temperature using a Raman spectrometry system (Jobin-Yvon T64000, France). The hydrodynamic size agglomerates and charge measurements of ZnO NPs in distilled water (DW) were determined by dynamic light scattering (DLS) using the Zetasizer Nano ZS instrument (Malvern, Malvern, UK). Additionally, the specific surface areas of the NPs were measured by Brunauer-Emmett-Teller adsorption analysis on a Micromeritics ASAP 2010 M + C instrument (Micromeritics Co, GA, USA).

### Animals and treatment

Male Wistar rats (4 weeks old) weighing 130–150 g were purchased from the Animal Center of Southern Medical University (Guangzhou, China). One week prior to beginning the experiment, the rats were housed under controlled environmental conditions (23 ± 1 °C room temperature, 60 ± 10% relative humidity and a 12 h/12 h light/dark cycle). Rodent diet and water were provided ad libitum.

The rats were randomly allocated into three groups with comparable weights: the control group, the ZnO NPs tongue instillation group and the ZnO NPs gavage group. The exposure procedure referred to the intranasal instillation procedure in other intranasal instillation studies [37, 87]. The concentration of ZnO NPs exposure was 50 mg/kg body weight (BW), which was based on the doses of oral administration in other animal models [15, 33]. ZnO NPs were dispersed in DW (50 mg/mL) and sonicated for at least 30 min before the administration. Rats of the control and ZnO NP instillation groups were weighed and anaesthetized under 1% pentobarbital sodium via intraperitoneal injection (48 mg/kg) before instillation. The rats were held in the lateral position, and their tongues were pulled from the corner of their mouth. Then, a 50 mg/mL suspension of ZnO NPs (50 mg/kg BW) was instilled onto the surface of the tongue with a microsyringe, and the control group was instilled with an equal amount of DW. The instillation procedure lasted approximately one hour, and the tongue was then rinsed with DW to clean the remainder of the NPs. For the ZnO gavage group, the rats were treated with a 50 mg/mL suspension of ZnO NPs (50 mg/kg BW) by oral gavage and anaesthetized in the same manner 1 h later to eliminate the effects of anaesthesia. These protocols were performed every other day for 30 days.

After 30 days of treatment, animals from each group (*n* = 5) were anaesthetized and exsanguinated. After blood collection, the animals were perfused with 100 mL of ice-cold saline (0.9% NaCl in deionized water with 10 U/mL heparin) and fixed in ice-cold 4% paraformaldehyde. Fixed tissue samples were analysed by histopathology examination and immunohistochemistry (IHC). The remaining animals were sacrificed to collect tissues, including the brain (cerebellum, brainstem, cerebral cortex and hippocampus), nerves (CT and glossopharyngeal nerve) and blood, which were extracted for the analysis of Zn content (*n* = 6), cytokine release (*n* = 6) and quantitative real-time PCR (qRT­PCR; *n* = 6), respectively.

### ICP-MS elemental analysis of tissues and blood

The levels of Zn in the blood or sub-brain regions were quantified by inductively coupled plasma mass spectrometry (ICP-MS). The collected tissues and blood were pre-digested in concentrated nitric acid overnight, completely digested after the addition of H_2_O_2_ and heated to 160 °C. The solutions were heated again to 120 °C until the remaining nitric acid evaporated to nearly dryness. The resulting solutions were finally diluted to 2 mL with 1% nitric acid and 0.1% Triton-100 and analysed using ICP-MS. The detection limit of ICP-MS was 0.05–0.8 mg/L Zn for method validation.

### Inflammatory responses in the rat brain

The gene expression levels of TNF-α, IL-1β, IL-6, IL-10, IFNG and NOS2 were determined by qRT­PCR. The expression level of each target gene was normalized to the GAPDH mRNA content and analysed using the 2^-∆∆CT^ method. The primers for qRT­PCR are shown in Table 2.

**Table 2:** Primer sequences specific for rats used in the qRT-PCR analysis

Gene	Forward primer	Reverse primer
TNF-α	ACTGAACTTCGGGGTGATTG	GCTTGGTGGTTTGCTACGAC
IL-1β	TGCAAGTGTCTGAAGCAGCTA	ATCTGGACAGCCCAAGTCAAG
IL-6	ATTGTATGAACAGCGATGATGC	CCAGAGCAGATTTTCAATAGGC
IL-10	CCAGTCAGCCAGACCCACAT	GGGGCATCACTTCTACCAGG
IFNG	AGCAACAGTAAAGCAAAAAAGGAT	ACCGACTCCTTTTCCGCTTC
NOS2	AGCCGCACCACCCTCCTTG	CACACAGTTTGGTCTGGCGAA
GAPDH	AGTGCCAGCCTCGTCTCATA	GATGGTGATGGGTTTCCCGT

Frozen brain samples were homogenized, and RNA was extracted using TRIzol reagent (Sigma­Aldrich, USA) according to the manufacturer’s instructions. The concentration and purity of the RNA was measured at 260 and 280 nm using a microspectrophotometer (NanoDrop, Thermo Scientific, USA). Synthesized complementary DNA (cDNA) was reverse transcribed from the RNA samples (1000 ng) using a reverse transcription (RT) reagent kit (PrimeScript RT Master Mix, TaKaRa, Japan). RT-PCR was carried out using a Real-time PCR kit (SYBR Premix Ex Taq, TaKaRa, Japan) and analysed on a LightCycler480 Sequence Detector System (Roche, Switzerland).

Cerebral cortex levels of TNF-α and IL-1β in the three groups were measured by an enzyme linked immunosorbent assay (ELISA) kit specific for rats (Biolegend, San Diego, CA). The assays were performed strictly according to the manufacturer’s instructions. Absorbance was read using a microplate reader (M5, Molecular Devices, USA) at a wavelength of 450 nm.

### Histopathological and immunohistochemical examination of the tongue and brain

The fixed tongue and brain tissues were dehydrated in a graded series of ethanol and xylene solutions, embedded in paraffin blocks and sectioned at a 4 μm thickness using a microtome. Haematoxylin & eosin (H&E) and Nissl staining were performed using standard laboratory procedures. Finally, the sections were observed, and photos were acquired using a light microscope (Bx51, Olympus, Japan).

Furthermore, IHC analysis was also carried out to identify specific neurotoxic effects. Astrocyte and microglial cell activation in the brain sections was detected by glial fibrillary acidic protein (GFAP; Cell Signaling Technology (CST), USA) and CD11b (Abcam, USA), respectively. Anti-P_2_X purinoceptor 7 (P_2_RX_7_; Abcam) was used to label the ionotropic P_2_X_7_ receptor. Photographs of positive staining (brown) were captured in randomly selected fields of both the prefrontal cortex and the hippocampus and counted by ImageJ software.

### Cell culture

The BV2 and PC12 cell lines were purchased from CBCAS (Cell Bank of the Chinese Academy of Sciences, Shanghai, People’s Republic of China) and maintained in DMEM containing 10% foetal bovine serum, 100 U/mL penicillin and 100 μg/mL streptomycin at 37 °C in a 5% CO_2_ humidified incubator. The culture medium was replaced every other day, and cells were passaged upon reaching approximately 80% confluence.

ZnO NPs were diluted to 100 μg/mL from a 1 mg/mL stock solution in the culture medium and sonicated for at least 30 min to prevent aggregation before the cells were treated.

For inhibition experiments, cells were preincubated for 1 h with the following substances: A839977 (200 nM; MCE, USA), a P_2_RX_7_ receptor antagonist, and BAPTA-AM (20 μM; MCE, USA), an intracellular Ca^2+^ chelator, before ZnO NPs treatment.

### MTT assay and LDH measurement

Cell survival rates following treatment with different concentrations of ZnO NPs (5, 10, 15, 20, 30, 40, 50 and 60 μg/mL) were evaluated using the MTT assay. Briefly, BV2 and PC12 cells were seeded onto 96-well culture plates at a density of 5 × 10^3^ cells/well and allowed to attach overnight. Then, the cells were exposed to various concentrations of ZnO NPs for 1, 3, 6, 12, 24 and 48 h. Cell viability was evaluated using the MTT assay (*n* = 6), and the absorbance was measured using a microplate reader at a wavelength of 570 nm.

Lactate dehydrogenase (LDH) leakage is based on the measurement of LDH activity in the extracellular medium, which releases from damaged cells. After exposure to ZnO NPs, the culture medium was removed and centrifuged at 3000 rpm for 5 min to obtain the cell-free supernatant. The LDH activity in the medium was determined using a commercial LDH kit (Nanjing Jiancheng, Nanjing, China) according to the manufacturer’s protocol, and the absorption was measured using a microplate reader at a wavelength of 450 nm.

### Inflammatory responses at the cellular level

BV2 and PC12 cells were seeded on 6-well plates, treated with ZnO NPs for a specific amount of time, and rinsed twice with PBS before TRIzol reagent was added to extract the RNA. The subsequent steps were performed in accordance with the qRT­PCR detection method described for the in vivo study. The primers used for qRT­PCR in PC12 and BV2 cells are shown in Tables 2 and 3, respectively.

**Table 3:** Primer sequences specific for mice used in the qRT-PCR analysis

Gene	Forward primer	Reverse primer
TNF-α	GGCACTCCCCCAAAAGATGG	GCTCCTCCACTTGGTGGTTT
IL-1β	TGCCACCTTTTGACAGTGATG	TGATACTGCCTGCCTGAAGC
IL-6	TTGCCTTCTTGGGACTGATG	CAGAATTGCCATTGCACAACTC
IL-10	TGGCCCAGAAATCAAGGAGCAT	CACTCTTCACCTGCTCCACTGC
IFNG	AGCAAGGCGAAAAAGGATGC	TCATTGAATGCTTGGCGCTG
NOS2	CGCTCTAGTGAAGCAAAGCC	TGATGGACCCCAAGCAAGAC
GAPDH	CTTCGGGCCACGCTAATCTC	ATGAAGGGGTCGTTGATGGC

TNF-α and IL-1β in the culture medium after ZnO NPs treatment were measured by ELISA using the same protocol as that used to measure cytokine release in vivo.

### Ultrastructural changes in the cells

For the TEM examination, BV2 and PC12 cells were seeded on 6-well plates and incubated with 30 μg/mL ZnO NPs for amount of time, and rinsed twice with PBS before collection and then removed from plate by scraping. After fixing with glutaraldehyde, cells were post-fixed in 1% osmium tetroxide, dehydrated, and embedded in epon. Thin sections were collected on uncoated copper grids, stained with uranyl acetate and lead citrate and then examined with TEM instrument.

### Calcium imaging

After incubating with 30 μg/mL ZnO NPs for 1, 3, 6 and 12 h in BV2 and PC12 cells, intracellular calcium levels were analyzed using calcium kit (Fluo4-AM, Dojindo, Japan) according to the manufacturer’s protocol. The fluorescence of calcium were observed under an automatic fluorescence microscope (BX63, Olympus, Japan).

### Western bolt

After treatmenting with ZnO NPs, cells were washed twice with PBS, and then RIPA lysis buffer (Beyotime, Haimen, China) with protease inhibitor and phosphatase inhibitors was added to extract the protein. The bicinchoninic acid (BCA) protein assay (Pierce BCA Protein Assay Kit; Thermo Fisher Scientifc, USA) was used to determine protein concentration. An equal amount of protein was separated using 8% SDS-PAGE. The proteins were transferred to polyvinylidene difluoride membranes (Merck Millipore, Darmstadt, Germany) and blocked with TBST containing 5% skim milk for 1 h at room temperature and then incubated overnight at 4 °C with specific antibodies including: anti-P_2_X_7_ (1:1000, abcam), NF-κB p65, phospho-NF-κB p65, p44/42 MAPK, phospho- p44/42 MAPK, p38 MAPK, phospho-p38 MAPK (1:1000, CST) and GAPDH (1:1000, Proteintech, USA). The blots were then washed by TBST, exposed to HRP-conjugated secondary antibodies (1:1000, CST, USA) for 1 h, and the antigen-antibody complex was detected with chemiluminescent HRP substrate (Millipore) by a chemiluminescence measuring instrument (Tanon 5200, Shanghai, China).

### Statistical analysis

All analyzes were performed in SPSS 19.0 software and the results are expressed as the means ± SD. All data were statistically analyzed by analysis of variance (ANOVA). A homogeneity-of-variance test was performed, and Least—Significant Difference (LSD) and Dunnett’s T3 tests were used when equal variance was assumed or not. *P*-values less than 0.05 were considered significant.

## Additional files

Additional file 1: **Figure S5.** Zn level and the release of cytokines in tissue and blood. The concentrations of Zn in the nerves (CT and glossopharyngeal nerve), sub-brain regions (A) and blood (B). The Zn levels of nerve and sub-brain regions in the tongue instillation group were significantly higher than other four groups, and there were no significant increases in the blood Zn concentrations compared with that in other groups. The concentration of TNF-α(C) and IL-1β(D) were analyzed in the rat brain of five groups. The concentrations of TNF-α and IL-1β were significantly increased in ZnO NPs tongue instillation group compared with other four groups. Results shown as means ± SD, compare with background group, *P < 0.05, **P < 0.01. (*n* = 6). (TIF 30505 kb)

Additional file 2: **Figure S1.** The inflammatory responses to ZnO NPs in PC12 cells. The mRNA relative values of cytokines gene expressions in PC12 cells (A). The gene expression levels of TNF-α, IL-6 and NOS2 were significant up-regulation after ZnO NPs exposure at 1, 3, and 6 h. The concentration of TNF-α and IL-1β in PC12 cells culture supernatants were performed by ELISA analysis (B). TNF-α was significantly increased after ZnO NPs stimulation in PC12 cells. Results shown as means ± SD from three independent experiments, compare with control group, *P < 0.05, **P < 0.01. Abbreviations: ZnO: zinc oxide nanoparticles; SD: Standard deviation. (TIF 46023 kb)

Additional file 3: **Figure S2.** The expression of involved mechanism of inflammation after treatment with ZnO NPs. PC12 cells were treated with ZnO NPs at a dose of 0, 5, 10, 15, 20 or 30 μg/mL for 1, 3, 6 or 12 h. Total proteins were extracted, and the levels of NF-κB, ERK and p38 signaling pathway molecules were analyzed via Western Blot(A). The gray value was semiquantitative as shown in histogram (B). The phosphorylation levels of NF-κB, ERK and p38 were significantly increase after ZnO NPs treatment. Results shown as means ± SD from three independent experiments, compare with control group, *P < 0.05, **P < 0.01. Abbreviations: ZnO: zinc oxide nanoparticles; SD: Standard deviation. (TIF 32619 kb)

Additional file 4: **Figure S3.** Effect of ZnO NPs on the intracellular Ca^2+^, expression of P_2_X_7_ receptor and ultrastructure changes in PC12 cells. PC12 cells were treated with ZnO NPs (30 μg/mL) for 1, 3, 6 or 12 h, cells were stained with Ca^2+^ indicator Fluo4-AM (green) and DAPI (blue). Scale bar represents 200 μm. Compare the mean fluorescence intensity of Ca^2+^ measured in PC12 cells (B), and an obvious increase in the Ca^2+^ fluorescence(green) was observed in a time-dependent manner. The expression of P_2_X_7_ receptor was increased in a dose-dependent manner after treating with ZnO NPs in PC12 cells at doses of 0, 5, 10, 15, 20 or 30 μg/mL for 1, 3, 6 or 12 h (C). The dilatational endoplasmic reticulum and the empty and swollen mitochondria were observed after ZnO NPs (30 μg/mL) for 6 h treatment in PC12 cells (D). The yellow arrows show the dilatational endoplasmic reticulum and the red arrows show the swollen mitochondria. Results shown as means ± SD from three independent experiments, compare with control group, *P < 0.05, **P < 0.01. Abbreviations: cont: control; ZnO: zinc oxide nanoparticles; P_2_X_7_: P_2_X purinoceptor 7; DAPI: 4,6-diamino-2-phenyl indole; SD: Standard deviation. (TIF 34215 kb)

Additional file 5: **Figure S4.** ZnO NPs-induced inflammation is mediated by calcium-dependent pathways in PC12 cells. PC12 cells were preincubated for 1 h with A839977 (200 nM) and BAPTA-AM (20 μM) before ZnO NPs treatment. After the cells were treated with ZnO NPs at a dose of 30 μg/ml for 6 h. Summary of cell viability (A), TNF-α (B) and IL-1β (C) release and the expression of proinflammatory genes(F). The concentration of Ca^2+^ in PC12 cells, cells were stained with Ca^2+^ indicator Fluo4-AM (green) and DAPI (blue) (D). Scale bar represents 200 μm. Compare the mean fluorescence intensity of Ca^2+^ measured in PC12 cells (G). Total proteins of PC12 cells were extracted, and the levels of P_2_X_7_, NF-κB, ERK and p38 signaling pathway molecules were analyzed via Western Blot (E). The gray value was semiquantitative as shown in histogram (H). The 30 μg/mL ZnO NPs-induced cytotoxicity, increase in proinflammatory cytokine release and NF-κB, ERK and p38 phosphorylation was significantly inhibited by BAPTA-AM. Results shown as means ± SD from three independent experiments, compare with control group, *P < 0.05, **P < 0.01. Abbreviations: cont: control; ZnO: zinc oxide nanoparticles; P_2_X_7_: P_2_X purinoceptor 7; DAPI: 4,6-diamino-2-phenyl indole SD: Standard deviation. (TIF 41705 kb)

## Abbreviations

AD: Alzheimer’s disease
ALS: Amyotrophic lateral sclerosis
BBB: Blood brain barrier
BET: Brunner emmet teller
Ca^2+^: Calcium ions
CNS: Central nervous system
CT: Chorda tympani
DLS: Dynamic light scattering
FTLD: Frontotemporal lobar dementia
GFAP: Glial fibrillary acidic protein
H&E: Haematoxylin & eosin
HRP: Horseradish peroxidase
ICP: Inductively coupled plasma
LDH: Lactate dehydrogenase
NO: Nitric oxide
P_2_RX_7_: P_2_X purinoceptor 7
PD: Parkinson’s disease
PM: Particulate material
ROS: Reactive oxygen species
TEM: Transmission electron microscopy
TiO_2_ NPs: Titanium dioxide nanoparticles
ZnO NPs: Zinc oxide nanoparticles

## References

[1] Frohlich E, Roblegg E (2012). Models for oral uptake of nanoparticles in consumer products. Toxicology.

[2] Javidi M, Zarei M, Naghavi N, Mortazavi M, Nejat AH (2014). Zinc oxide nano-particles as sealer in endodontics and its sealing ability. Contemp Clin Dent.

[3] Memarzadeh K, Sharili AS, Huang J, Rawlinson SC, Allaker RP (2015). Nanoparticulate zinc oxide as a coating material for orthopedic and dental implants. J Biomed Mater Res A.

[4] Li CH, Shen CC, Cheng YW, Huang SH, Wu CC, Kao CC (2012). Organ biodistribution, clearance, and genotoxicity of orally administered zinc oxide nanoparticles in mice. Nanotoxicology.

[5] Okada Y, Tachibana K, Yanagita S, Takeda K (2013). Prenatal exposure to zinc oxide particles alters monoaminergic neurotransmitter levels in the brain of mouse offspring. J Toxicol Sci.

[6] Xiaoli F, Junrong W, Xuan L, Yanli Z, Limin W, Jia L (2017). Prenatal exposure to nanosized zinc oxide in rats: neurotoxicity and postnatal impaired learning and memory ability. Nanomedicine (Lond).

[7] Modrzynska J, Berthing T, Ravn-Haren G, Jacobsen NR, Weydahl IK, Loeschner K (2018). Primary genotoxicity in the liver following pulmonary exposure to carbon black nanoparticles in mice. Part Fibre Toxicol.

[8] Paek HJ, Lee YJ, Chung HE, Yoo NH, Lee JA, Kim MK (2013). Modulation of the pharmacokinetics of zinc oxide nanoparticles and their fates in vivo. Nanoscale.

[9] Kao YY, Cheng TJ, Yang DM, Wang CT, Chiung YM, Liu PS (2012). Demonstration of an olfactory bulb-brain translocation pathway for ZnO nanoparticles in rodent cells in vitro and in vivo. J Mol Neurosci.

[10] Gao L, Yang ST, Li S, Meng Y, Wang H, Lei H (2013). Acute toxicity of zinc oxide nanoparticles to the rat olfactory system after intranasal instillation. J Appl Toxicol.

[11] Gonzalez-Maciel A, Reynoso-Robles R, Torres-Jardon R, Mukherjee PS, Calderon-Garciduenas L (2017). Combustion-derived nanoparticles in key brain target cells and organelles in young urbanites: culprit hidden in plain sight in Alzheimer's disease development. J Alzheimers Dis.

[12] Patchin ES, Anderson DS, Silva RM, Uyeminami DL, Scott GM, Guo T (2016). Size-dependent deposition, translocation, and microglial activation of inhaled silver nanoparticles in the rodent nose and brain. Environ Health Perspect.

[13] Ahmad E, Feng Y, Qi J, Fan W, Ma Y, He H (2017). Evidence of nose-to-brain delivery of nanoemulsions: cargoes but not vehicles. Nanoscale.

[14] Liu Q, Shen Y, Chen J, Gao X, Feng C, Wang L (2012). Nose-to-brain transport pathways of wheat germ agglutinin conjugated PEG-PLA nanoparticles. Pharm Res.

[15] Aijie C, Huimin L, Jia L, Lingling O, Limin W, Junrong W (2017). Central neurotoxicity induced by the instillation of ZnO and TiO2 nanoparticles through the taste nerve pathway. Nanomedicine (Lond).

[16] Ransohoff RM (2016). How neuroinflammation contributes to neurodegeneration. Science.

[17] Wang Y, Xiong L, Tang M (2017). Toxicity of inhaled particulate matter on the central nervous system: neuroinflammation, neuropsychological effects and neurodegenerative disease. J Appl Toxicol.

[18] Simon-Vazquez R, Lozano-Fernandez T, Davila-Grana A, Gonzalez-Fernandez A (2016). Analysis of the activation routes induced by different metal oxide nanoparticles on human lung epithelial cells. Future Sci OA.

[19] Wu W, Samet JM, Peden DB, Bromberg PA (2010). Phosphorylation of p65 is required for zinc oxide nanoparticle-induced interleukin 8 expression in human bronchial epithelial cells. Environ Health Perspect.

[20] Saptarshi SR, Feltis BN, Wright PF, Lopata AL (2015). Investigating the immunomodulatory nature of zinc oxide nanoparticles at sub-cytotoxic levels in vitro and after intranasal instillation in vivo. J Nanobiotechnology.

[21] Chuang HC, Juan HT, Chang CN, Yan YH, Yuan TH, Wang JS (2014). Cardiopulmonary toxicity of pulmonary exposure to occupationally relevant zinc oxide nanoparticles. Nanotoxicology.

[22] Tian L, Lin B, Wu L, Li K, Liu H, Yan J (2015). Neurotoxicity induced by zinc oxide nanoparticles: age-related differences and interaction. Sci Rep.

[23] Grosse S, Stenvik J, Nilsen AM (2016). Iron oxide nanoparticles modulate lipopolysaccharide-induced inflammatory responses in primary human monocytes. Int J Nanomedicine.

[24] Sun Q, Ishii T, Kanehira K, Sato T, Taniguchi A (2017). Uniform TiO2 nanoparticles induce apoptosis in epithelial cell lines in a size-dependent manner. Biomater Sci.

[25] Prach M, Stone V, Proudfoot L (2013). Zinc oxide nanoparticles and monocytes: impact of size, charge and solubility on activation status. Toxicol Appl Pharmacol.

[26] Simon M, Saez G, Muggiolu G, Lavenas M, Le Trequesser Q, Michelet C (2017). In situ quantification of diverse titanium dioxide nanoparticles unveils selective endoplasmic reticulum stress-dependent toxicity. Nanotoxicology.

[27] Bhattacharya D, Santra CR, Ghosh AN, Karmakar P (2014). Differential toxicity of rod and spherical zinc oxide nanoparticles on human peripheral blood mononuclear cells. J Biomed Nanotechnol.

[28] Palomaki J, Valimaki E, Sund J, Vippola M, Clausen PA, Jensen KA (2011). Long, needle-like carbon nanotubes and asbestos activate the NLRP3 inflammasome through a similar mechanism. ACS Nano.

[29] Li Y, Zhang W, Niu J, Chen Y (2012). Mechanism of photogenerated reactive oxygen species and correlation with the antibacterial properties of engineered metal-oxide nanoparticles. ACS Nano.

[30] Chatterjee N, Eom HJ, Choi J (2014). A systems toxicology approach to the surface functionality control of graphene-cell interactions. Biomaterials.

[31] Haase A, Dommershausen N, Schulz M, Landsiedel R, Reichardt P, Krause BC (2017). Genotoxicity testing of different surface-functionalized SiO2, ZrO2 and silver nanomaterials in 3D human bronchial models. Arch Toxicol.

[32] Xu M, Zhu J, Wang F, Xiong Y, Wu Y, Wang Q (2016). Improved in vitro and in vivo biocompatibility of graphene oxide through surface modification: poly(acrylic acid)-functionalization is superior to PEGylation. ACS Nano.

[33] Cho WS, Kang BC, Lee JK, Jeong J, Che JH, Seok SH (2013). Comparative absorption, distribution, and excretion of titanium dioxide and zinc oxide nanoparticles after repeated oral administration. Part Fibre Toxicol.

[34] Shrivastava R, Raza S, Yadav A, Kushwaha P, Flora SJ (2014). Effects of sub-acute exposure to TiO2, ZnO and Al2O3 nanoparticles on oxidative stress and histological changes in mouse liver and brain. Drug Chem Toxicol.

[35] Wang B, Feng W, Wang M, Wang T, Gu Y, Zhu M (2008). Acute toxicological impact of nano- and submicro-scaled zinc oxide powder on healthy adult mice. J Nanopart Res.

[36] Baek M, Chung HE, Yu J, Lee JA, Kim TH, Oh JM (2012). Pharmacokinetics, tissue distribution, and excretion of zinc oxide nanoparticles. Int J Nanomedicine.

[37] Wang J, Liu Y, Jiao F, Lao F, Li W, Gu Y (2008). Time-dependent translocation and potential impairment on central nervous system by intranasally instilled TiO(2) nanoparticles. Toxicology.

[38] Wang Y, Wang B, Zhu MT, Li M, Wang HJ, Wang M (2011). Microglial activation, recruitment and phagocytosis as linked phenomena in ferric oxide nanoparticle exposure. Toxicol Lett.

[39] Nance E, Zhang F, Mishra MK, Zhang Z, Kambhampati SP, Kannan RM (2016). Nanoscale effects in dendrimer-mediated targeting of neuroinflammation. Biomaterials.

[40] Alawieyah Syed Mortadza S, Sim JA, Neubrand VE, Jiang LH (2018). A critical role of TRPM2 channel in Abeta42 -induced microglial activation and generation of tumor necrosis factor-alpha. Glia.

[41] Sumbria RK, Boado RJ, Pardridge WM (2012). Brain protection from stroke with intravenous TNFalpha decoy receptor-Trojan horse fusion protein. J Cereb Blood Flow Metab.

[42] Becher B, Spath S, Goverman J (2017). Cytokine networks in neuroinflammation. Nat Rev Immunol.

[43] Liu J, Kang Y, Zheng W, Song B, Wei L, Chen L (2017). Ion-shedding zinc oxide nanoparticles induce microglial BV2 cell proliferation via the ERK and Akt signaling pathways. Toxicol Sci.

[44] Fukui H, Horie M, Endoh S, Kato H, Fujita K, Nishio K (2012). Association of zinc ion release and oxidative stress induced by intratracheal instillation of ZnO nanoparticles to rat lung. Chem Biol Interact.

[45] Rizzuto R, Pozzan T (2003). When calcium goes wrong: genetic alterations of a ubiquitous signaling route. Nat Genet.

[46] Tang M, Wang M, Xing T, Zeng J, Wang H, Ruan DY (2008). Mechanisms of unmodified CdSe quantum dot-induced elevation of cytoplasmic calcium levels in primary cultures of rat hippocampal neurons. Biomaterials.

[47] George S, Pokhrel S, Xia T, Gilbert B, Ji Z, Schowalter M (2010). Use of a rapid cytotoxicity screening approach to engineer a safer zinc oxide nanoparticle through iron doping. ACS Nano.

[48] Ikeda M, Tsuno S, Sugiyama T, Hashimoto A, Yamoto K, Takeuchi K (2013). Ca(2+) spiking activity caused by the activation of store-operated ca(2+) channels mediates TNF-alpha release from microglial cells under chronic purinergic stimulation. Biochim Biophys Acta.

[49] Brough D, Le Feuvre RA, Wheeler RD, Solovyova N, Hilfiker S, Rothwell NJ, et al. Ca2+ stores and Ca2+ entry differentially contribute to the release of IL-1 beta and IL-1 alpha from murine macrophages. J Immunol. 170(6):3029–36. 10.4049/jimmunol.170.6.3029 12626557

[50] Koizumi S, Shigemoto-Mogami Y, Nasu-Tada K, Shinozaki Y, Ohsawa K, Tsuda M (2007). UDP acting at P2Y6 receptors is a mediator of microglial phagocytosis. Nature.

[51] Farber K, Kettenmann H (2006). Functional role of calcium signals for microglial function. Glia.

[52] Hide I, Tanaka M, Inoue A, Nakajima K, Kohsaka S, Inoue K (2000). Extracellular ATP triggers tumor necrosis factor-alpha release from rat microglia. J Neurochem.

[53] Hoffmann A, Kann O, Ohlemeyer C, Hanisch UK, Kettenmann H (2003). Elevation of basal intracellular calcium as a central element in the activation of brain macrophages (microglia): suppression of receptor-evoked calcium signaling and control of release function. J Neurosci.

[54] Huang CC, Aronstam RS, Chen DR, Huang YW (2010). Oxidative stress, calcium homeostasis, and altered gene expression in human lung epithelial cells exposed to ZnO nanoparticles. Toxicol In Vitro.

[55] Amadio S, Parisi C, Piras E, Fabbrizio P, Apolloni S, Montilli C, et al. Modulation of P2X7 receptor during inflammation in multiple sclerosis. Front Immunol. 2017;8. 10.3389/fimmu.2017.01529 PMC5694754 29187851

[56] Miras-Portugal M. Teresa, Sebastián-Serrano Álvaro, de Diego García Laura, Díaz-Hernández Miguel (2017). Neuronal P2X7 Receptor: Involvement in Neuronal Physiology and Pathology. The Journal of Neuroscience.

[57] Di Virgilio F, Dal Ben D, Sarti AC, Giuliani AL, Falzoni S (2017). The P2X7 receptor in infection and inflammation. Immunity.

[58] Kurashima Y, Amiya T, Nochi T, Fujisawa K, Haraguchi T, Iba H (2012). Extracellular ATP mediates mast cell-dependent intestinal inflammation through P2X7 purinoceptors. Nat Commun.

[59] Shieh CH, Heinrich A, Serchov T, van Calker D, Biber K (2014). P2X7-dependent, but differentially regulated release of IL-6, CCL2, and TNF-alpha in cultured mouse microglia. Glia.

[60] Shiratori M, Tozaki-Saitoh H, Yoshitake M, Tsuda M, Inoue K (2010). P2X7 receptor activation induces CXCL2 production in microglia through NFAT and PKC/MAPK pathways. J Neurochem.

[61] Panenka W, Jijon H, Herx LM, Armstrong JN, Feighan D, Wei T (2001). P2X7-like receptor activation in astrocytes increases chemokine monocyte chemoattractant protein-1 expression via mitogen-activated protein kinase. J Neurosci.

[62] Albalawi F, Lu W, Beckel JM, Lim JC, McCaughey SA, Mitchell CH (2017). The P2X7 receptor primes IL-1beta and the NLRP3 Inflammasome in astrocytes exposed to mechanical strain. Front Cell Neurosci.

[63] Wei W, Ryu JK, Choi HB, McLarnon JG (2008). Expression and function of the P2X(7) receptor in rat C6 glioma cells. Cancer Lett.

[64] Dong Z, Saikumar P, Weinberg JM, Venkatachalam MA (2006). Calcium in cell injury and death. Annu Rev Pathol.

[65] Jain AK, Senapati VA, Singh D, Dubey K, Maurya R, Pandey AK (2017). Impact of anatase titanium dioxide nanoparticles on mutagenic and genotoxic response in Chinese hamster lung fibroblast cells (V-79): the role of cellular uptake. Food Chem Toxicol.

[66] Zhang T, Tang M, Zhang S, Hu Y, Li H, Zhang T (2017). Systemic and immunotoxicity of pristine and PEGylated multi-walled carbon nanotubes in an intravenous 28 days repeated dose toxicity study. Int J Nanomedicine.

[67] Gilmore TD (2006). Introduction to NF-kappaB: players, pathways, perspectives. Oncogene.

[68] Kim EK, Choi EJ (2015). Compromised MAPK signaling in human diseases: an update. Arch Toxicol.

[69] Simon-Vazquez R, Lozano-Fernandez T, Davila-Grana A, Gonzalez-Fernandez A (2016). Metal oxide nanoparticles interact with immune cells and activate different cellular responses. Int J Nanomedicine.

[70] Nishanth RP, Jyotsna RG, Schlager JJ, Hussain SM, Reddanna P (2011). Inflammatory responses of RAW 264.7 macrophages upon exposure to nanoparticles: role of ROS-NFkappaB signaling pathway. Nanotoxicology.

[71] Romoser AA, Chen PL, Berg JM, Seabury C, Ivanov I, Criscitiello MF (2011). Quantum dots trigger immunomodulation of the NFkappaB pathway in human skin cells. Mol Immunol.

[72] Tiwari R, Singh RD, Khan H, Gangopadhyay S, Mittal S, Singh V (2017). Oral subchronic exposure to silver nanoparticles causes renal damage through apoptotic impairment and necrotic cell death. Nanotoxicology.

[73] Park JW, Lee IC, Shin NR, Jeon CM, Kwon OK, Ko JW (2016). Copper oxide nanoparticles aggravate airway inflammation and mucus production in asthmatic mice via MAPK signaling. Nanotoxicology.

[74] Bianchi MG, Allegri M, Chiu M, Costa AL, Blosi M, Ortelli S (2017). Lipopolysaccharide adsorbed to the bio-Corona of TiO2 nanoparticles powerfully activates selected pro-inflammatory transduction pathways. Front Immunol.

[75] Hong F, Wu N, Ge Y, Zhou Y, Shen T, Qiang Q (2016). Nanosized titanium dioxide resulted in the activation of TGF-beta/Smads/p38MAPK pathway in renal inflammation and fibration of mice. J Biomed Mater Res A.

[76] Chen J, Zhang J, Cao J, Xia Z, Gan J (2016). Inflammatory MAPK and NF-kappaB signaling pathways differentiated hepatitis potential of two agglomerated titanium dioxide particles. J Hazard Mater.

[77] Brown DM, Donaldson K, Borm PJ, Schins RP, Dehnhardt M, Gilmour P (2004). Calcium and ROS-mediated activation of transcription factors and TNF-alpha cytokine gene expression in macrophages exposed to ultrafine particles. Am J Lung Cell Mol Physiol.

[78] Scherbart AM, Langer J, Bushmelev A, van Berlo D, Haberzettl P, van Schooten FJ (2011). Contrasting macrophage activation by fine and ultrafine titanium dioxide particles is associated with different uptake mechanisms. Part Fibre Toxicol.

[79] Yamamoto S, Shimizu S, Kiyonaka S, Takahashi N, Wajima T, Hara Y (2008). TRPM2-mediated Ca2+influx induces chemokine production in monocytes that aggravates inflammatory neutrophil infiltration. Nat Med.

[80] Ratner AJ, Bryan R, Weber A, Nguyen S, Barnes D, Pitt A (2001). Cystic fibrosis pathogens activate Ca2+−dependent mitogen-activated protein kinase signaling pathways in airway epithelial cells. J Biol Chem.

[81] Wei L, Wang J, Chen A, Jia L, Feng X, Shao L (2017). Involvement of PINK1/parkin-mediated mitophagy in ZnO nanoparticle-induced toxicity in BV-2 cells. Int J Nanomedicine.

[82] Li YB, Zhang QH, Chen Z, He ZJ, Yi GH (2015). Oxidized low-density lipoprotein attenuated desmoglein 1 and desmocollin 2 expression via LOX-1/ca(2+)/PKC-beta signal in human umbilical vein endothelial cells. Biochem Biophys Res Commun.

[83] Shukla A, Ramos-Nino M, Mossman B (2003). Cell signaling and transcription factor activation by asbestos in lung injury and disease. Int J Biochem Cell Biol.

[84] Moore ED, Kooshki M, Metheny-Barlow LJ, Gallagher PE, Robbins ME (2013). Angiotensin-(1-7) prevents radiation-induced inflammation in rat primary astrocytes through regulation of MAP kinase signaling. Free Radic Biol Med.

[85] Gilbert DF, Stebbing MJ, Kuenzel K, Murphy RM, Zacharewicz E, Buttgereit A (2016). Store-operated ca(2+) entry (SOCE) and purinergic receptor-mediated ca(2+) homeostasis in murine bv2 microglia cells: early cellular responses to ATP-mediated microglia activation. Front Mol Neurosci.

[86] Traves PG, Pimentel-Santillana M, Carrasquero LM, Perez-Sen R, Delicado EG, Luque A (2013). Selective impairment of P2Y signaling by prostaglandin E2 in macrophages: implications for Ca2+−dependent responses. J Immunol.

[87] Zhang L, Bai R, Liu Y, Meng L, Li B, Wang L (2012). The dose-dependent toxicological effects and potential perturbation on the neurotransmitter secretion in brain following intranasal instillation of copper nanoparticles. Nanotoxicology.

