# Supplementary figures and images for: Neuroinflammation is induced by tongue-instilled ZnO nanoparticles via the Ca2+-dependent NF-κB and MAPK pathways
Source: Part Fibre Toxicol. 2018 Oct 19;15:39. doi: 10.1186/s12989-018-0274-0 (PMC6194560; doi:10.1186/s12989-018-0274-0)

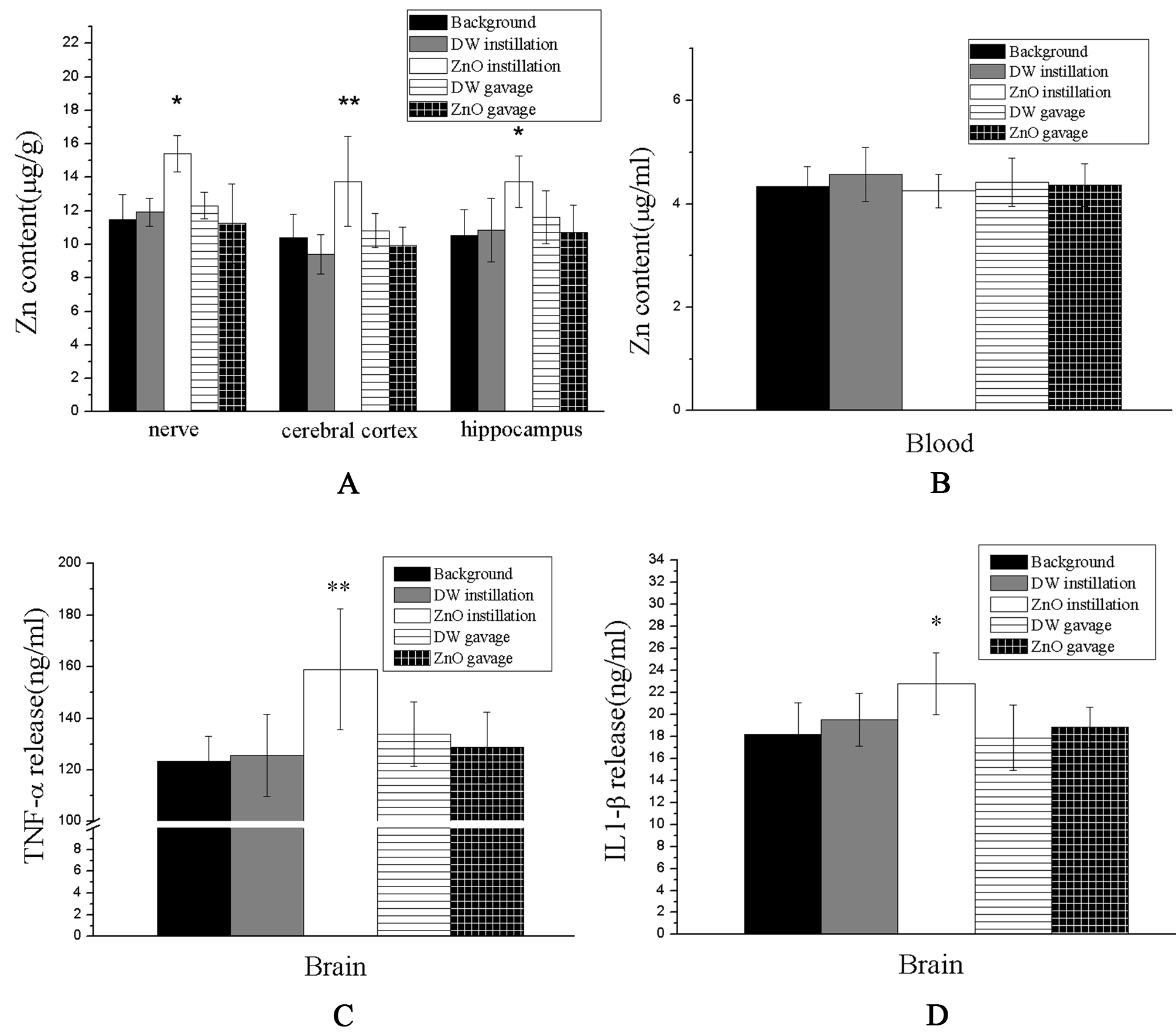

Supplement: Supplementary file 1 — Figure S5. Zn level and the release of cytokines in tissue and blood. The concentrations of Zn in the nerves (CT and glossopharyngeal nerve), sub-brain regions (A) and blood (B). The Zn levels of nerve and sub-brain regions in the tongue instillation group were significantly higher than other four groups, and there were no significant increases in the blood Zn concentrations compared with that in other groups. The concentration of TNF-α(C) and IL-1β(D) were analyzed in the rat brain of five groups. The concentrations of TNF-α and IL-1β were significantly increased in ZnO NPs tongue instillation group compared with other four groups. Results shown as means ± SD, compare with background group, *P < 0.05, **P < 0.01. (n = 6). (TIF 30505 kb) [file 12989_2018_274_MOESM1_ESM.tif]

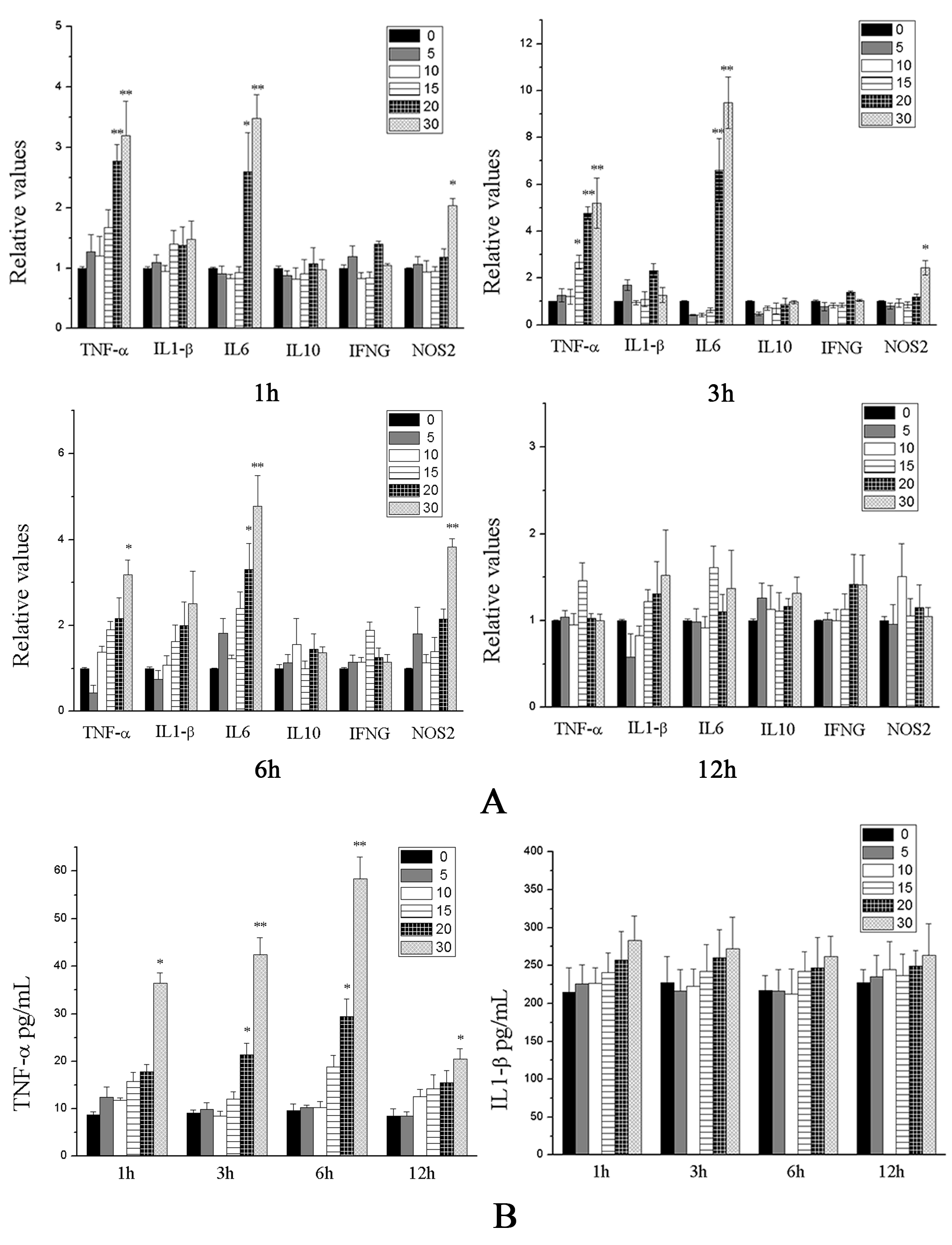

Supplement: Supplementary file 2 — Figure S1. The inflammatory responses to ZnO NPs in PC12 cells. The mRNA relative values of cytokines gene expressions in PC12 cells (A). The gene expression levels of TNF-α, IL-6 and NOS2 were significant up-regulation after ZnO NPs exposure at 1, 3, and 6 h. The concentration of TNF-α and IL-1β in PC12 cells culture supernatants were performed by ELISA analysis (B). TNF-α was significantly increased after ZnO NPs stimulation in PC12 cells. Results shown as means ± SD from three independent experiments, compare with control group, *P < 0.05, **P < 0.01. Abbreviations: ZnO: zinc oxide nanoparticles; SD: Standard deviation. (TIF 46023 kb) [file 12989_2018_274_MOESM2_ESM.tif]

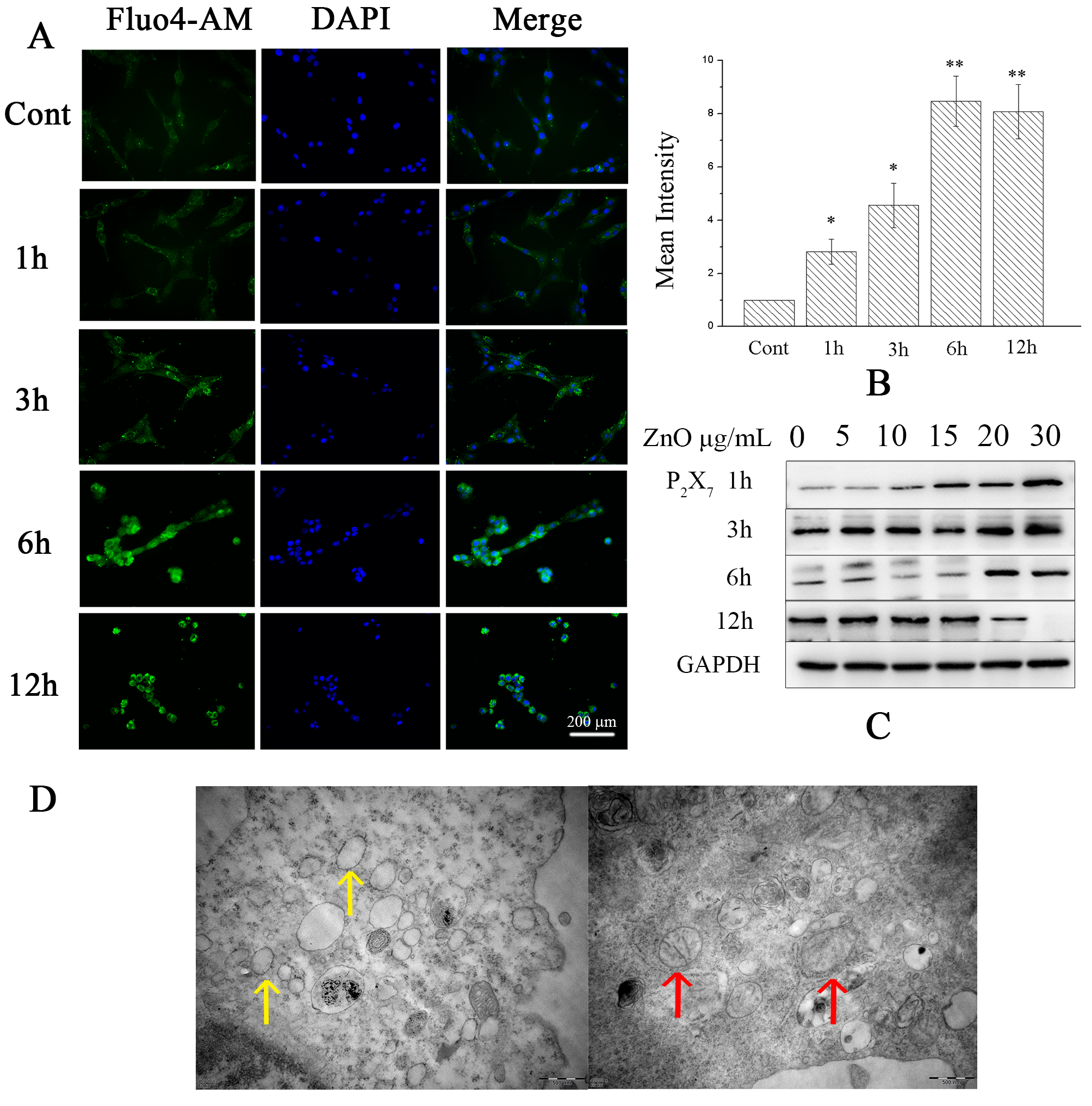

Supplement: Supplementary file 3 — Figure S2. The expression of involved mechanism of inflammation after treatment with ZnO NPs. PC12 cells were treated with ZnO NPs at a dose of 0, 5, 10, 15, 20 or 30 μg/mL for 1, 3, 6 or 12 h. Total proteins were extracted, and the levels of NF-κB, ERK and p38 signaling pathway molecules were analyzed via Western Blot(A). The gray value was semiquantitative as shown in histogram (B). The phosphorylation levels of NF-κB, ERK and p38 were significantly increase after ZnO NPs treatment. Results shown as means ± SD from three independent experiments, compare with control group, *P < 0.05, **P < 0.01. Abbreviations: ZnO: zinc oxide nanoparticles; SD: Standard deviation. (TIF 32619 kb) [file 12989_2018_274_MOESM3_ESM.tif]

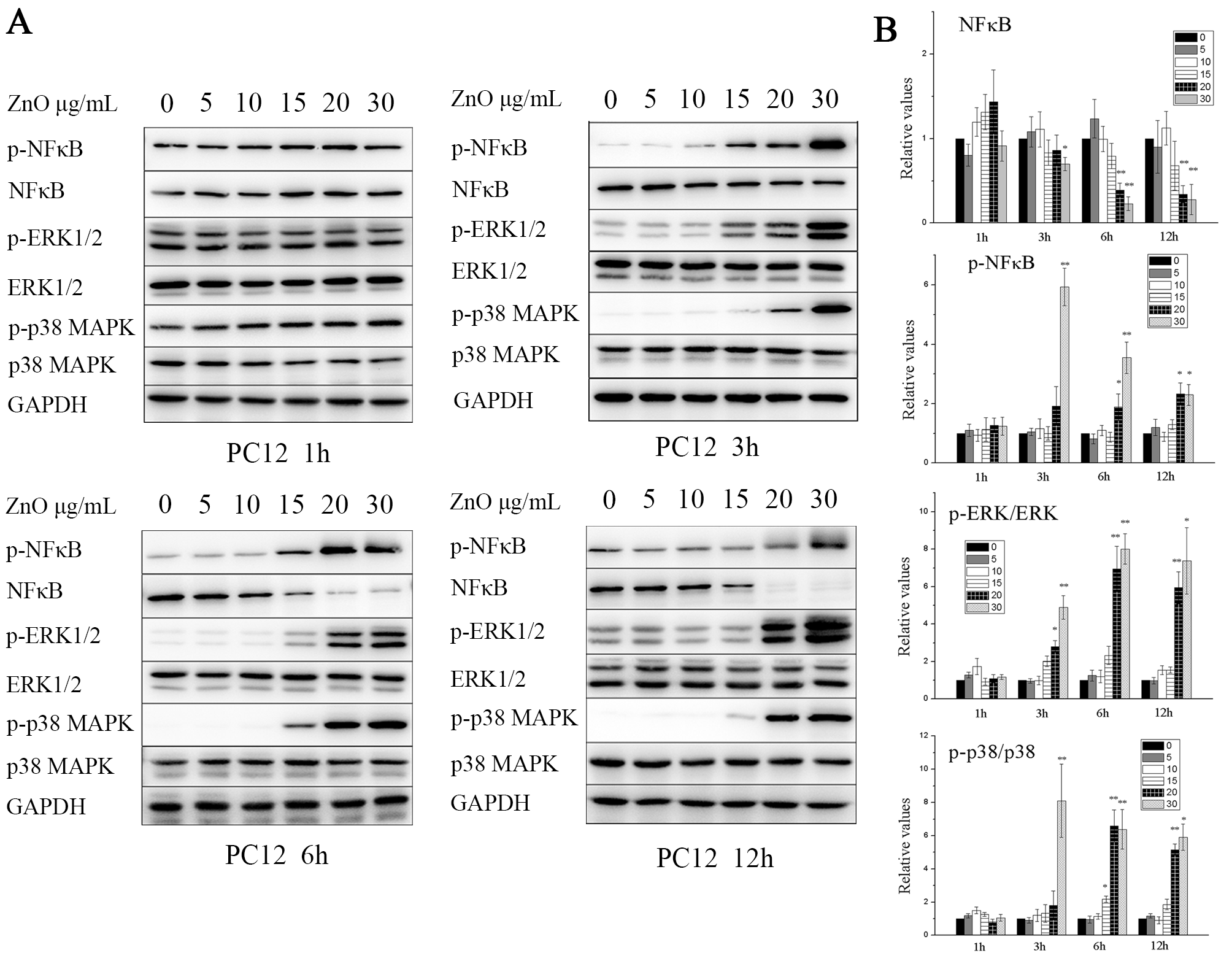

Supplement: Supplementary file 4 — Figure S3. Effect of ZnO NPs on the intracellular Ca2+, expression of P2X7 receptor and ultrastructure changes in PC12 cells. PC12 cells were treated with ZnO NPs (30 μg/mL) for 1, 3, 6 or 12 h, cells were stained with Ca2+ indicator Fluo4-AM (green) and DAPI (blue). Scale bar represents 200 μm. Compare the mean fluorescence intensity of Ca2+ measured in PC12 cells (B), and an obvious increase in the Ca2+ fluorescence(green) was observed in a time-dependent manner. The expression of P2X7 receptor was increased in a dose-dependent manner after treating with ZnO NPs in PC12 cells at doses of 0, 5, 10, 15, 20 or 30 μg/mL for 1, 3, 6 or 12 h (C). The dilatational endoplasmic reticulum and the empty and swollen mitochondria were observed after ZnO NPs (30 μg/mL) for 6 h treatment in PC12 cells (D). The yellow arrows show the dilatational endoplasmic reticulum and the red arrows show the swollen mitochondria. Results shown as means ± SD from three independent experiments, compare with control group, *P < 0.05, **P < 0.01. Abbreviations: cont: control; ZnO: zinc oxide nanoparticles; P2X7: P2X purinoceptor 7; DAPI: 4,6-diamino-2-phenyl indole; SD: Standard deviation. (TIF 34215 kb) [file 12989_2018_274_MOESM4_ESM.tif]

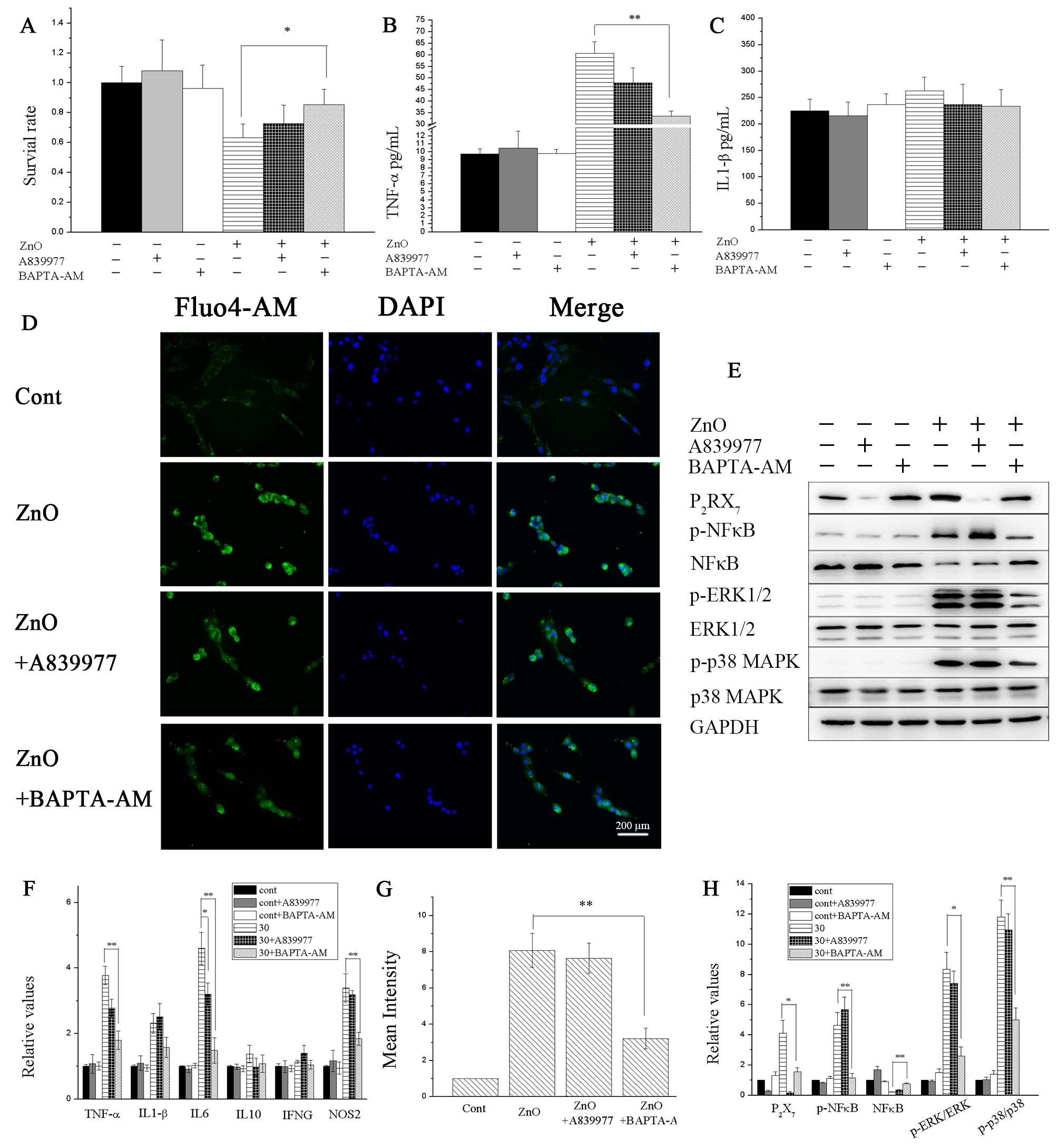

Supplement: Supplementary file 5 — Figure S4. ZnO NPs-induced inflammation is mediated by calcium-dependent pathways in PC12 cells. PC12 cells were preincubated for 1 h with A839977 (200 nM) and BAPTA-AM (20 μM) before ZnO NPs treatment. After the cells were treated with ZnO NPs at a dose of 30 μg/ml for 6 h. Summary of cell viability (A), TNF-α (B) and IL-1β (C) release and the expression of proinflammatory genes(F). The concentration of Ca2+ in PC12 cells, cells were stained with Ca2+ indicator Fluo4-AM (green) and DAPI (blue) (D). Scale bar represents 200 μm. Compare the mean fluorescence intensity of Ca2+ measured in PC12 cells (G). Total proteins of PC12 cells were extracted, and the levels of P2X7, NF-κB, ERK and p38 signaling pathway molecules were analyzed via Western Blot (E). The gray value was semiquantitative as shown in histogram (H). The 30 μg/mL ZnO NPs-induced cytotoxicity, increase in proinflammatory cytokine release and NF-κB, ERK and p38 phosphorylation was significantly inhibited by BAPTA-AM. Results shown as means ± SD from three independent experiments, compare with control group, *P < 0.05, **P < 0.01. Abbreviations: cont: control; ZnO: zinc oxide nanoparticles; P2X7: P2X purinoceptor 7; DAPI: 4,6-diamino-2-phenyl indole SD: Standard deviation. (TIF 41705 kb) [file 12989_2018_274_MOESM5_ESM.tif]
